# Integrin intra-heterodimer affinity inversely correlates with integrin activatability

**DOI:** 10.1016/j.celrep.2021.109230

**Published:** 2021-06-08

**Authors:** Guangyu Sun, Emilie Guillon, Scott A. Holley

**Affiliations:** 1 Department of Molecular, Cellular and Developmental Biology, Yale University, 260 Whitney Avenue, New Haven, CT 06520, USA; 2 Lead contact

## Abstract

Integrins are heterodimeric cell surface receptors composed of an α and β subunit that mediate cell adhesion to extracellular matrix proteins such as fibronectin. We previously studied integrin α5β1 activation during zebrafish somitogenesis, and in the present study, we characterize the integrin αV fibronectin receptors. Integrins are activated via a conformational change, and we perform single-molecule biophysical measurements of both integrin activation via fluorescence resonance energy transfer (FRET)-fluorescence lifetime imaging microscopy (FLIM) and integrin intra-heterodimer stability via fluorescence cross-correlation spectroscopy (FCCS) in living embryos. We find that integrin heterodimers that exhibit robust cell surface expression, including αVβ3, αVβ5, and αVβ6, are never activated in this *in vivo* context, even in the presence of fibronectin matrix. In contrast, activatable integrins, such as integrin αVβ1, and alleles of αVβ3, αVβ5, αVβ6 that are biased to the active conformation exhibit poor cell surface expression and have a higher intra-heterodimer dissociation constant (K_D_). These observations suggest that a weak integrin intra-heterodimer affinity decreases integrin cell surface stability and increases integrin activatability.

## In brief

Sun et al. examine fibronectin receptor biophysics in live embryos and find that many of these integrins are never activated along the zebrafish somite boundary. Integrins α5β1 and αVβ1 are activated, but integrins αVβ3, αVβ5, and αVβ6 exhibit no activity. Integrin intra-heterodimer affinity appears to determine how readily an integrin is activated.

## Graphical Abstract

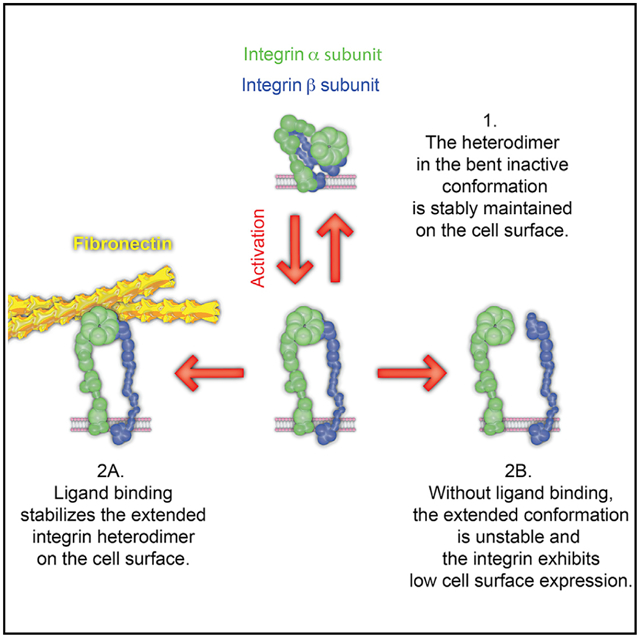

## INTRODUCTION

Integrins are a major class of adhesion receptors with mammals having 18 α subunits and 8 β subunits that form 24 different integrin heterodimers (Hynes, 2002). When activated by either the intracellular (inside-out signaling) or extracellular (outside-in signaling) environment, integrins undergo a conformational change that increases their ligand binding affinity (Campbell and Humphries, 2011). Integrin heterodimers are assembled in the endoplasmic reticulum and activated on the cell surface (Hynes, 2002; Lenter and Vestweber, 1994; Tiwari et al., 2011). In cell culture, most cell surface integrins are in a bent-closed conformation, as this form is both energetically favored and recycled more rapidly than active integrins (Arjonen et al., 2012; Li and Springer, 2018; Li et al., 2017). Integrin activation is affected by ligand specificity, matrix rigidity, and tensile force (Bachmann et al., 2020; Elosegui-Artola et al., 2016; Friedland et al., 2009).

Integrins α5β1 and αVβ3 bind the extracellular matrix (ECM) protein fibronectin (FN) by recognizing the Arg-Gly-Asp (RGD) motif (Hynes, 2002; Schwarzbauer and DeSimone, 2011). Integrins α5β1 and αVβ3 display both unique and redundant roles in focal adhesion regulation, ECM assembly, and mechano-signal transduction (Morgan et al., 2009; Roca-Cusachs et al., 2009; Schiller et al., 2013; Takahashi et al., 2007; Yang et al., 1999). For example, integrin αVβ3 more stably resides in focal contacts while α5β1 is more dynamic and translocates away from focal contacts along actin filaments to mediate further fibronectin matrix assembly (Pankov et al., 2000; Rossier et al., 2012). In signal transduction, integrin α5β1, but not αVβ3, can activate RhoA in some cell types (Danen et al., 2002). In mechano-transduction, α5β1 responds in a biphasic manner to mechanical load due to its catch bond with fibronectin, but αV-class integrins do not exhibit this behavior (Strohmeyer et al., 2017). Notably, most of these studies were performed in cell culture, whereas little is known about molecular dynamics of integrins *in vivo*.

**Figure 1.**
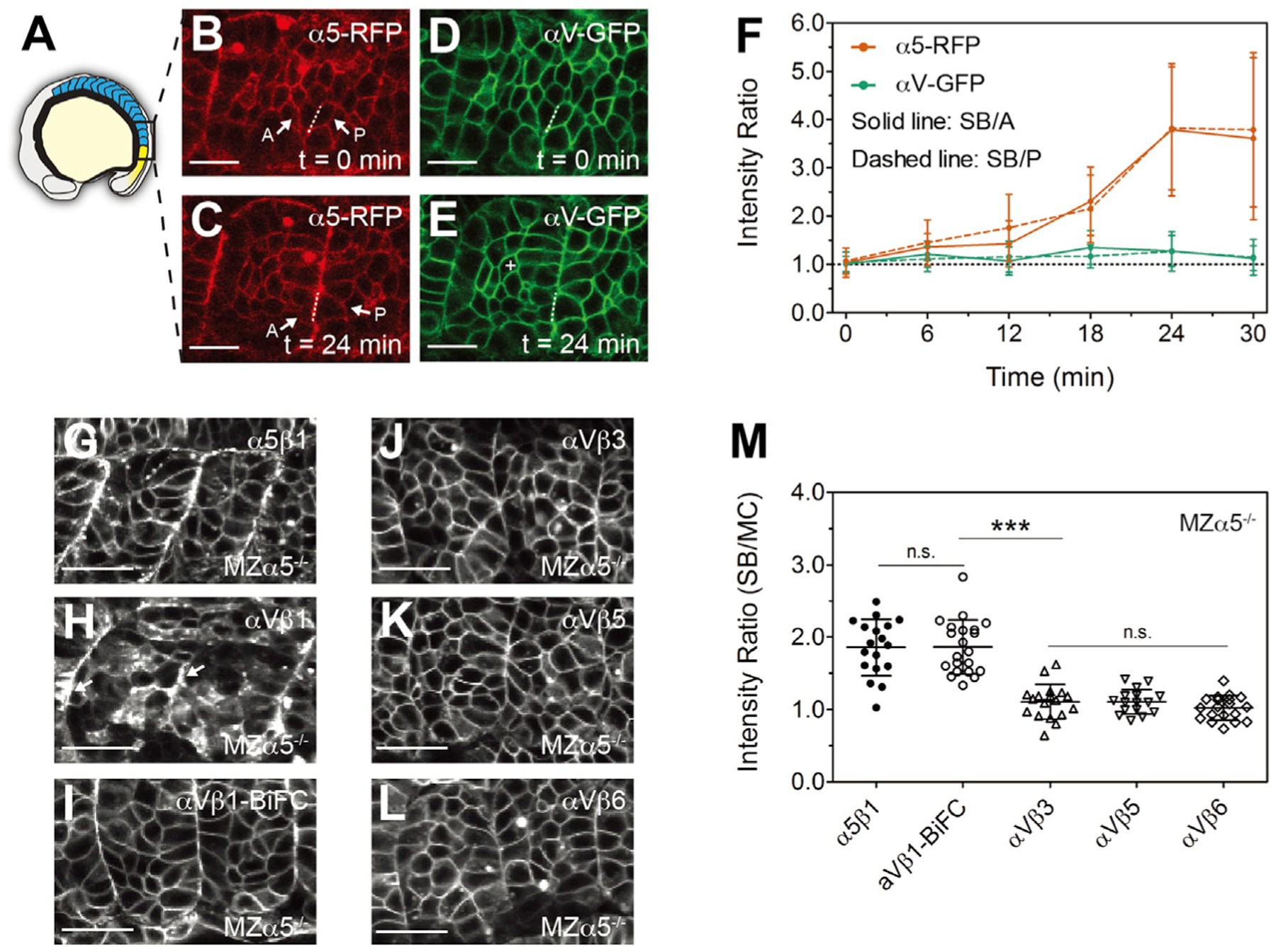
Integrins α5β1 and αVβ1, but not αVβ3, αVβ5, and αVβ6, cluster along somite boundaries (A) Illustration of a zebrafish embryo highlighting the somites (blue) and presomitic mesoderm (yellow). (B–E) Confocal images of integrin α5-RFP (B and C) and αV-GFP (D and E) in wild-type (WT) embryos. As the somite boundary (SB) forms, α5 clusters to the basal side (dashed lines) of the anterior (A) and posterior (P) boundary cells (arrows). The white cross in (E) denotes a mesenchymal cell (MC) within a somite. Scale bars, 20 μm. (F) Basal/apical ratio of integrin intensity in anterior (SB/A, solid line) and posterior (SB/P, dashed line) boundary cells. Data are mean ± SD from n = 15 cell pairs in six embryos. (G–L) Integrin α5-Aquamarine (Aqm) and αV-Aqm co-expressed with different integrin β subunits tagged with mCitrine (mCit) in developing somites of MZα5^−/−^ embryos. (G) α5β1, (H) αVβ1, (I) αVβ1-BiFC (bimolecular fluorescence complementation, used to increase heterodimer stability), (J) αVβ3, (K) αVβ5, and (L) αVβ6. Arrows in (H) indicate clustering on the somite border. Scale bars, 30 μm. (M) Clustering quantification via the SB/MC intensity ratio. Details of ROI selection shown in Figure S1A. α5β1, n = 18 measurements (12 embryos); αVβ1-BiFC, n = 21 (13 embryos); αVβ3, n = 18 (8 embryos); αVβ5, n = 16 (14 embryos); αVβ6, n = 19 (9 embryos). Data are mean ± SD. ***p < 0.0001; n.s., not significant (two-sided t test). See also Figure S1.

During zebrafish somitogenesis, both integrins α5 and αV are required for fibronectin matrix assembly along somite boundaries (SBs) (Dray et al., 2013; Jülich et al., 2005; Koshida et al., 2005). Somites are mesodermal segments containing precursors of the vertebrae and skeletal muscle (Figure 1A). Somite boundary formation entails a mesenchymal-to-epithelial transition by the cells along the boundary, with the basal sides forming the boundary and the apical sides adhering to mesenchymal cells within the core of the somite. We previously studied integrin α5β1 activation during zebrafish somitogenesis (Jülich et al., 2015). In this study, we sought to explore the function of αV integrins in this process. We quantify integrin biophysics in this *in vivo* context using fluorescence resonance energy transfer (FRET), fluorescence lifetime imaging microscopy (FLIM), and fluorescence cross-correlation spectroscopy (FCCS). We find that α5β1 and αVβ1 are activated during somite boundary formation by adopting the extended open conformation. Surprisingly, other reported RGD binding integrins, including αVβ3, αVβ5, and αVβ6, remain inactive despite the presence of fibronectin matrix. Furthermore, we find that activatable integrins display poor cell membrane stability, and FCCS reveals that these integrins have a lower intra-heterodimer affinity. Our results suggest that integrin intra-heterodimer affinity determines how readily an integrin is activated.

## RESULTS

### Integrins α5β1 and αVβ1, but not αVβ3, αVβ5, and αVβ6, cluster along somite boundaries

Activated integrins cluster in focal adhesions and along ECM fibrils, and activated integrin α5β1 clusters along the zebrafish somite boundary (Cluzel et al., 2005; Jülich et al., 2015; Jülich et al., 2009; Roca-Cusachs et al., 2009). Therefore, we first compared integrin α5 and αV clustering during fibronectin matrix assembly on somite boundaries in live zebrafish embryos expressing red fluorescent protein-tagged α5 (α5-RFP) and green fluorescent protein-tagged αV (αV-GFP). To improve αV-GFP cell surface expression, β3 mRNA was co-expressed, whereas α5-RFP effectively localized to the cell membrane by heterodimerization with endogenous β1. We performed time-lapse imaging of the forming somites (Figures 1B–1E) and quantified integrin clustering by calculating the basal/apical fluorescence intensity ratio in somite boundary cells on both the anterior (SB/A) and posterior (SB/P) sides of the border. We determined when integrin clustering reached a plateau (t = 24 min, Figures 1C and 1E) and retrospectively plotted the rate of clustering starting when boundary cells began to differentiate from the presomitic mesoderm (PSM) (t = 0 min, Figures 1B, 1D, and 1F). Integrin α5β1 clusters until the intensity ratio increases almost 4-fold (SB/A = 3.6 ± 1.7 and SB/P = 3.8 ± 1.6, n = 15). The anterior and posterior boundary cells exhibit no difference in clustering. Surprisingly, integrin αVβ3 never clusters on the somite boundary. These results suggest that, unlike α5β1, αVβ3 is not activated on the somite border even in the presence of a fibronectin matrix.

**Figure 2.**
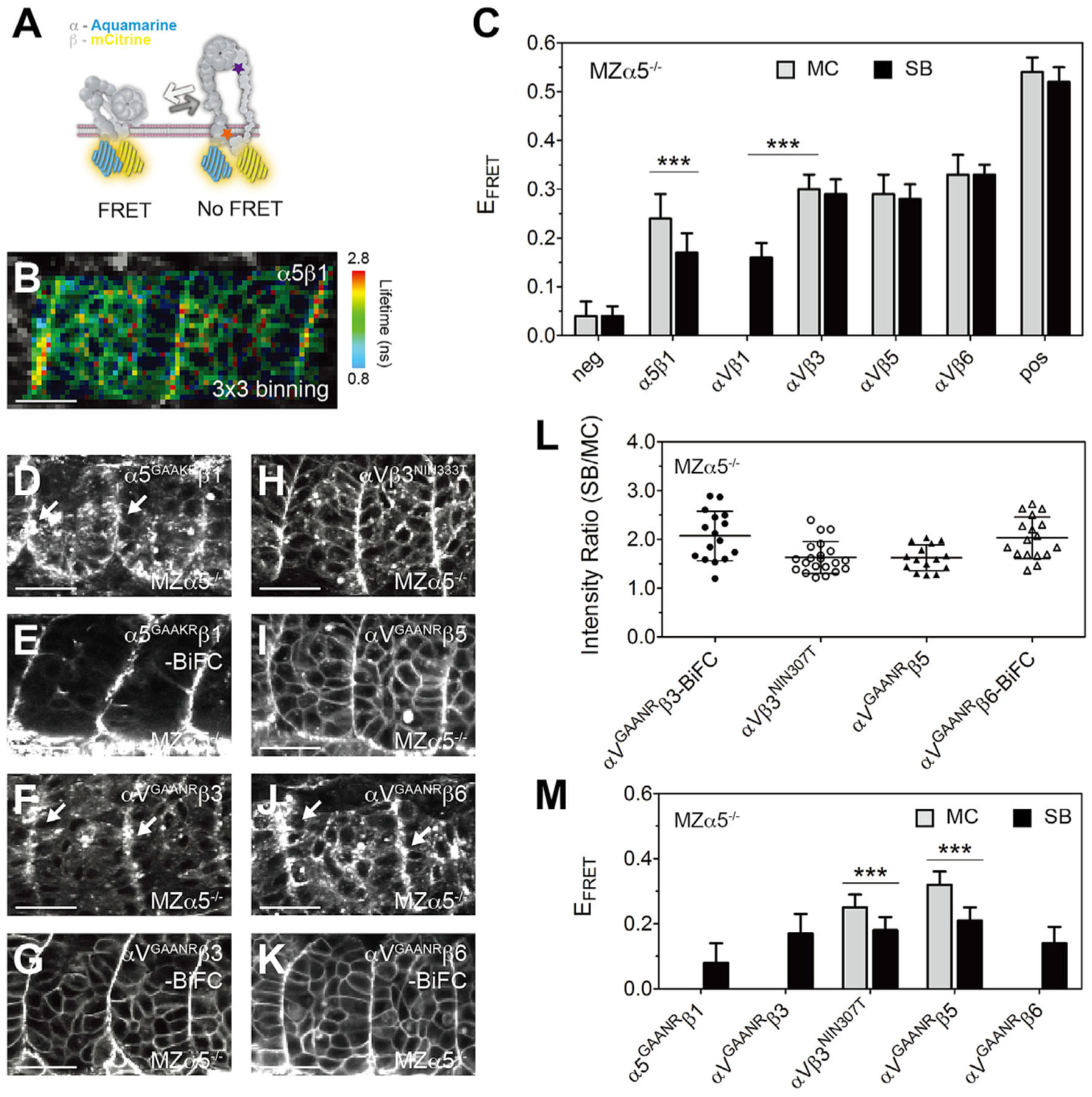
FRET-FLIM reveals heterodimer-specific activation on the somite boundary (A) Illustration of the FRET assay for the integrin conformation. The integrin α subunit cytoplasmic tail was tagged with Aqm as a FRET donor, and the β subunit was tagged with mCit as a FRET acceptor. When the cytoplasmic tails separate during integrin activation, FRET should be reduced. The locations of the α^GAAXR^ mutations (orange star) and the β3^NIN333T^ mutation (purple star) are indicated. (B) Heatmap of the fluorescence lifetime of integrin α5-Aqm co-expressed with β1-mCit (denoted α5β1). The raw image and lifetime distribution are shown in Figures S1C and S1D. Warm colors on the SB represent longer donor lifetimes, indicating weaker FRET and the active conformation. (C) FRET efficiency (E_FRET_) of different integrin heterodimers. Sample size is the same as in Figure 1M, except αVβ1, n = 20 measurements (12 embryos). (D–K) Activatable integrin alleles: α5^GAAKR^-Aqm co-expressed with β1-mCit (D), α5^GAAKR^β1-BiFC (E), αV^GAANR^-Aqm co-expressed with β3-mCit (F), αV^GAANR^β3-BiFC (G), αV-Aqm co-expressed with N-glycan wedge allele β3^NIN333T^-mCit (H), αV^GAANR^-Aqm co-expressed with β5-mCit (I), αV^GAANR^-Aqm co-expressed with β6-mCit (J), and αV^GAANR^β6-BiFC (K). White arrows in (D), (F), and (J) indicate clustering on the somite border. Scale bars, 30 μm. (L) Clustering quantification of activatable integrin alleles by the SB/MC intensity ratio. αV^GAANR^β3-BiFC, n = 16 (9 embryos); αVβ3^NIN333T^, n = 21 (15 embryos); αV^GAANR^β5, n = 15 (8 embryos); αV^GAANR^β6-BiFC, n = 17 (8 embryos). α5^GAAKR^β1-BiFC clustering cannot be measured because of the poor membrane expression in the mesenchyme. (M) E_FRET_ of activatable integrin alleles. Sample size is the same as in (L), except α5^GAAKR^β1, n = 18 (9 embryos); αV^GAANR^β3, n = 14 (9 embryos); and αV^GAANR^β6, n = 15 (7 embryos). E_FRET_ of αVβ1 (C), α5^GAAKR^β1, αV^GAANR^β3, and αV^GAANR^β6 (M) cannot be measured in the MC because of the poor membrane expression. (C and M) Data are mean ± SD. ***p < 0.0001, two-sided t test. All experiments are in MZα5^−/−^ embryos. See also Figure S1 and Table S1.

Using immunohistochemistry, we previously found that integrin α5 adopts the active open conformation when clustering on the somite border (Jülich et al., 2015). In this study, we sought to measure integrin conformation change during activation in living embryos using a FRET-FLIM assay (Kim et al., 2003). During FRET, the energy transfer from a donor fluorophore to an acceptor fluorophore results in a decrease in donor fluorescence lifetime, and thus FLIM provides a robust quantification of FRET. In this study, the integrin α subunit cytoplasmic tail was tagged with aquamarine (Aqm) as a FRET donor (Mérola et al., 2014), and the β subunit cytoplasmic tail was tagged with mCitrine (mCit) as a FRET acceptor. When the cytoplasmic tails separate during integrin activation, FRET should be reduced (Figure 2A). In addition to α5β1 and αVβ3, we also tested the other β subunits reported to heterodimerize with αV, including β1a, β1b, β5, β6, and β8 (Hynes, 2002). Of these, we never detected cell surface expression with β8. The two isoforms, β1a and β1b, exhibited similar results, and thus we show the data only for β1a (denoted as β1). To remove endogenous α5, these experiments were performed in maternal zygotic integrin α5 mutant (*MZα*5^−/−^) embryos (Figures 1G–1L) (Jülich et al., 2009).

We first quantified the clustering of the heterodimers on the somite boundary using the fluorescence intensity ratio of the somite boundary to mesenchymal cells (SB/MCs). Instead of choosing cell pairs along the somite boundary, pixels in the somite boundary and pixels in the mesenchymal cells were separately binned for both fluorescence intensity analysis and fluorescence lifetime profile construction (Figures 1M, S1A, and S1B). Consistent with our time-lapse results (Figure 1F), integrin α5β1 clustered on the somite boundary (Figure 1G) (SB/MC ratio = 1.9 ± 0.4, n = 18) while αVβ3 did not (Figure 1J) (SB/MC ratio = 1.1 ± 0.2, n = 18). Moreover, neither αVβ5 nor αVβ6 clustered on the somite boundary, although similar to αVβ3, they exhibited strong cell surface expression (Figures 1K–1M). In contrast, integrin αVβ1 clustered on the somite boundary, but it displayed poor cell surface expression in the mesenchymal cells (Figure 1H). To quantify αVβ1 clustering, we used Venus YFP bimolecular fluorescence complementation (BiFC) to stabilize the heterodimer. In this assay, the cytoplasmic tails of the heterodimer are tagged with either an N-terminal or C-terminal half of Venus, and the reconstitution of the YFP in the heterodimer non-covalently links the α and β subunits. Importantly, this physical coupling is flexible enough to allow integrin α5β1-BiFC to adopt the active conformation and rescue the somite boundary defect in *MZα*5^−/−^ mutants, indicating that the BiFC-tagged integrin is functional (Jülich et al., 2015; Jülich et al., 2009). Integrin αVβ1-BiFC exhibited strong cell surface expression and clustered on the somite boundary comparably to α5β1 (Figures 1I and 1M). We also found that integrin αVβ1 and αVβ1-BiFC rescued posterior somite boundary defects in embryos lacking both α5 and αV (Figure S2). These data suggest that integrin αVβ1 is the only αV heterodimer functional in zebrafish somitogenesis despite exhibiting lower cell surface expression than that for αVβ3, αVβ5 and αVβ6.

### FRET-FLIM reveals heterodimer-specific activation on the somite boundary

Next, we examined integrin heterodimer conformational changes via FRET-FLIM. FRET efficiency (E_FRET_) is calculated from the donor’s lifetime (τ_D_) in the absence and presence of the acceptor. In the inactive state, integrin heterodimers are closed bent conformers and should produce a strong FRET signal reflected as a short τ_D_ and a high E_FRET_. When activated, integrin heterodimers adopt the extended open conformation and should exhibit lower FRET marked by an increased τ_D_ and a reduced E_FRET_. Lifetime imaging of integrin α5-Aqm co-expressed with β1-mCit (denoted α5β1) showed a τ_D_ increase on the somite boundary as visualized via a heatmap (Figures 2B, S1C, and S1D), indicating that this FRET-FLIM assay can capture α5β1 activation conformational change along the somite boundary.

E_FRET_ was then calculated for the somite boundary and mesenchymal cell areas after pixel binning. A positive control was an Aqm-mCit fusion tagged to integrin α5 (α5-Aqm-mCit), which presented an E_FRET_ of around 0.52 (Figure 2C; Table S1). A negative control was provided by co-expression of intracellular myristoylated membrane-anchored Aqm (mem-Aqm) and mem-mCit and exhibited an E_FRET_ of 0.04. The E_FRET_ of integrin α5β1 dropped significantly from 0.24 ± 0.05 on mesenchymal cells to 0.17 ± 0.04 on the somite boundary (n = 18, p < 0.0001), consistent with its activation. In contrast, integrins αVβ3, αVβ5, and αVβ6 showed no such E_FRET_ change. For αVβ1, E_FRET_ was not measured in the mesenchymal cells because of the poor cell surface expression (Figure 1H). However, integrin αVβ1 displayed significantly lower E_FRET_ on the somite boundary than did any other αV heterodimer and was comparable to α5β1 (Figure 2C). These data indicate that integrin αVβ1, but not αVβ3, αVβ5, or αVβ6, adopts the active open conformation along somite boundaries.

Integrin αVβ3 is known as one of the two primary fibronectin receptors, so it is surprising that it is never activated by fibronectin along the somite boundary. We examined two mechanisms that might explain this lack of activation. First, we previously found that N-cadherin, i.e., Cadherin 2 (Cdh2), represses activation of integrin α5β1 in the zebrafish paraxial mesoderm (Jülich et al., 2015). Thus, we compared α5β1 and αVβ3 activation in the *cdh2*^−/−^ mutant using the clustering and FRET-FLIM assays (Figures S1E and S1F). We observed a reduced E_FRET_ of αVβ3 in the *cdh2*^−/−^ mutant compared with wild-type (WT), although the effect was weaker than that observed for α5β1. Nonetheless, αVβ3 did not cluster or activate along the somite boundary. Thus, repression by Cdh2 does not explain the lack of αVβ3 activation. Second, we tested whether the deadbolt model explained αVβ3 inactivity. This model proposes that association between the β tail domain and βI head in integrin β3 lock αVβ3 in an inactive state (Xiong et al., 2003). However, we found that disrupting this connection (Gupta et al., 2007) did not induce αVβ3 clustering or activation on the somite boundary (Figures S1G–S1I).

To further examine the regulation of αVβ3, we generated two alleles expected to bias the heterodimer to the active conformation. The first is αV^GAANR^ in which the conserved GFFNR motif is changed to GAANR, which abolishes the salt bridge between the α and β subunit in the membrane-proximal cytoplasmic domain, leading to separation of the cytoplasmic domains (O’Toole et al., 1994; Zhu et al., 2009). The second allele is β3^NIN333T^, which introduces an N-linked glycosylation site that results in a “glycan wedge” in the hybrid I-like domain interface that stabilizes the extended active conformation (Eng et al., 2011; Luo et al., 2003). The αV^GAANR^ allele was also used to assay αV^GAANR^β5 and αV^GAANR^β6 activity.

Similar to integrin αVβ1, αV^GAANR^β3 and αV^GAANR^β6 showed poor cell surface expression in the mesenchyme but localized on the somite border, and thus we used BiFC for their clustering quantification (Figures 2F, 2G, 2J, and 2K). Integrins αV^GAANR^β5 and αVβ3^NIN333T^ also exhibited lower cell surface expression and more cytoplasmic localization than did αVβ3 (Figures 2H and 2J). Strikingly, all of these alleles clustered on the somite boundary with a SB/MC ratio similar to α5β1 and αVβ1 (Figure 2L). The FRET-FLIM assay showed that they also adopted the active conformation with reduced E_FRET_ on the somite boundary (Figure 2M). As expected, the activated allele integrin α5^GAAKR^β1 also showed reduced E_FRET_ on the somite border and exhibited very poor cell surface expression in the mesenchyme (Figures 2D, 2E, and 2M). Taken together, these data suggest that activation of integrin α5β1 and αVβ1 is more energetically favorable than for either αVβ3, αVβ5, or αVβ6 because the latter three integrins require mutations that destabilize the inactive conformation in order to be activated by fibronectin on the somite boundary.

### Integrins α5β1 and αVβ1 are the functional fibronectin receptors in zebrafish somitogenesis

**Figure 3.**
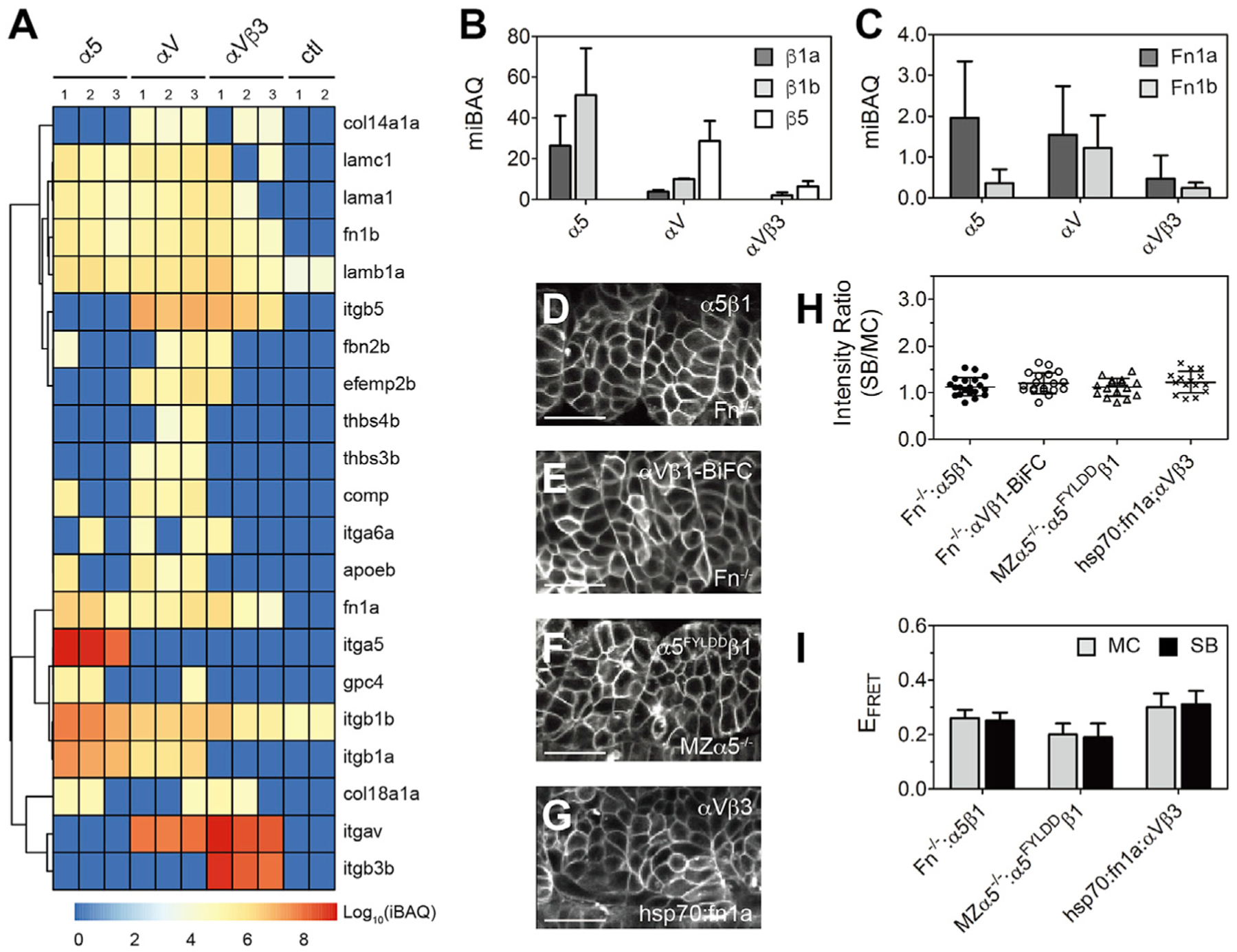
Integrins α5β1 and αVβ1 are the functional fibronectin (Fn) receptors during zebrafish somitogenesis (A) Integrins and ECM proteins co-immunoprecipitated with integrins α5, αV, or αVβ3 identified via mass spectroscopy. The intensity-based absolute quantification (iBAQ) from each replicate is color coded to show relative protein abundance. Hierarchical cluster analysis is shown as the dendrogram (see Tables S2 and S3 for protein names). Note that basement membrane ligand laminins (lama1, lamb1a, lamc1) are roughly equal in all three datasets, while thrombospondins (thbs3b, thbs4b) and cartilage oligomeric matrix protein (comp/thbs5) are found exclusively in the αV dataset. ctl, control (FLAG-tagged myristoylated membrane-anchored GFP [mem-GFP]). (B and C) Integrin β subunits (B) and Fn (C) quantification using median-normalized iBAQ (miBAQ). Bar indicates mean ± SD, n = 3. (D–G) Somite localization of integrin α5β1 (D) and αVβ1-BiFC (E) in Fn double-mutant Fn^−/−^ (*fn1a*^−/−^*;fn1b*^−/−^) embryos, ligand binding-deficient α5^FYLDD^β1 in MZα5^−/−^ embryos (F), and αVβ3 in heat shock promoter-driven Fn1a-mKikumi transgenic (*hsp70:fn1a*) embryos (G). Scale bars, 30 μm. (H and I) Clustering quantification (H) and E_FRET_ (I) of α5β1 in the absence of Fn, α5^FYLDD^ β1 in MZα5^−/−^ embryos, and αVβ3 exposed to extra Fn1a. Fn^−/−^: α5β1, n = 20 measurements (9 embryos); Fn^−/−^: αVβ1-BiFC, n = 19 (11 embryos); MZα5^−/−^: α5^FYLDD^ β1, n = 16 (8 embryos); *hsp70:fn1a*; αVβ3, n = 15 (7 embryos). Data are mean ± SD. See also Tables S1, S2, and S3 and Figure S2.

To better define the integrin-ECM protein network at the 10–13 somite stage of zebrafish development, we performed co-immunoprecipitation and mass spectrometry (MS)-based proteomics using FLAG-tagged integrins α5, αV, and αVβ3 expressed in *MZα*5^−/−^ embryos. In total, we identified 1,253 proteins (Tables S2 and S3). To estimate relative protein abundance, we used the intensity-based absolute quantification (iBAQ) algorithm (Schwanhäusser et al., 2011). Integrins β1a and β1b were enriched in the α5 dataset (Figures 3A and 3B). In the Integrin αV dataset, the primary β subunit was β5, followed by β1b and β1a. Integrin β3 was not detected, consistent with the report that β3 is not expressed until the 16–18 somite stage (Ablooglu et al., 2007). Co-injecting β3 mRNA with αV reduced the amount of β5 and β1 that was pulled down and concomitantly reduced the associated fibronectin 1a and fibronectin 1β (Fn1a and Fn1b) (Figures 3B and 3C). Altogether, the data suggest that αVβ1 is the primary αV integrin that engages in fibronectin matrix assembly along the somite boundary (Figure S2).

The MS data suggest that fibronectin is the primary ligand driving integrin activation at the somite boundary. To test this hypothesis, we examined α5β1 and αVβ1 clustering and activation in double homozygous fibronectin mutant embryos (*fn1a*^−/−^*;fn1b*^−/−^) (Guillon et al., 2020) (Figures 3D and 3E). Neither heterodimer clustered on the somite boundary, and FRET measurements indicate that α5β1 remained in the inactive closed conformation on the somite boundary (Figures 3H and 3I). Furthermore, a ligand binding-deficient α5^FYLDD^β1 (Jülich et al., 2009) expressed in *MZα*5^−/−^ embryos did not cluster or change conformation on the somite boundary despite the presence of fibronectin (Figures 3F, 3H, and 3I). Next, to test whether increasing fibronectin expression can drive αVβ3 activation, we performed experiments in transgenic zebrafish in which Fn1a is tagged with a photoconvertible protein mKikumeGR and expressed under the control of a heat-shock promoter (*hsp70:fn1a-mKIK*) (Guillon et al., 2020). After heat shock, we did not observe αVβ3 activation driven by the extra Fn1a expression (Figures 3G–3I). These results demonstrate that fibronectin drives activation of integrins α5β1 and αVβ1 and clustering along the somite boundary, but it does not activate αVβ3.

### Integrin intra-heterodimer affinity inversely correlates with integrin activatability

We found it curious that only integrins with relatively poor cell surface expression are activated along the somite boundary. Moreover, the C-terminal Venus BiFC tag, which provides an additional physical interaction between the heterodimer subunits, stabilizes integrin cell surface expression. These observations suggest that cell surface expression may be reduced by instability of the heterodimer. We hypothesized that integrins with a lower affinity between the α and β subunits are more easily activated, and that heterodimer dissociation reduces cell surface expression. If this hypothesis is correct, then activatable integrins should exhibit lower intra-heterodimer affinities than do un-activatable integrins.

**Figure 4.**
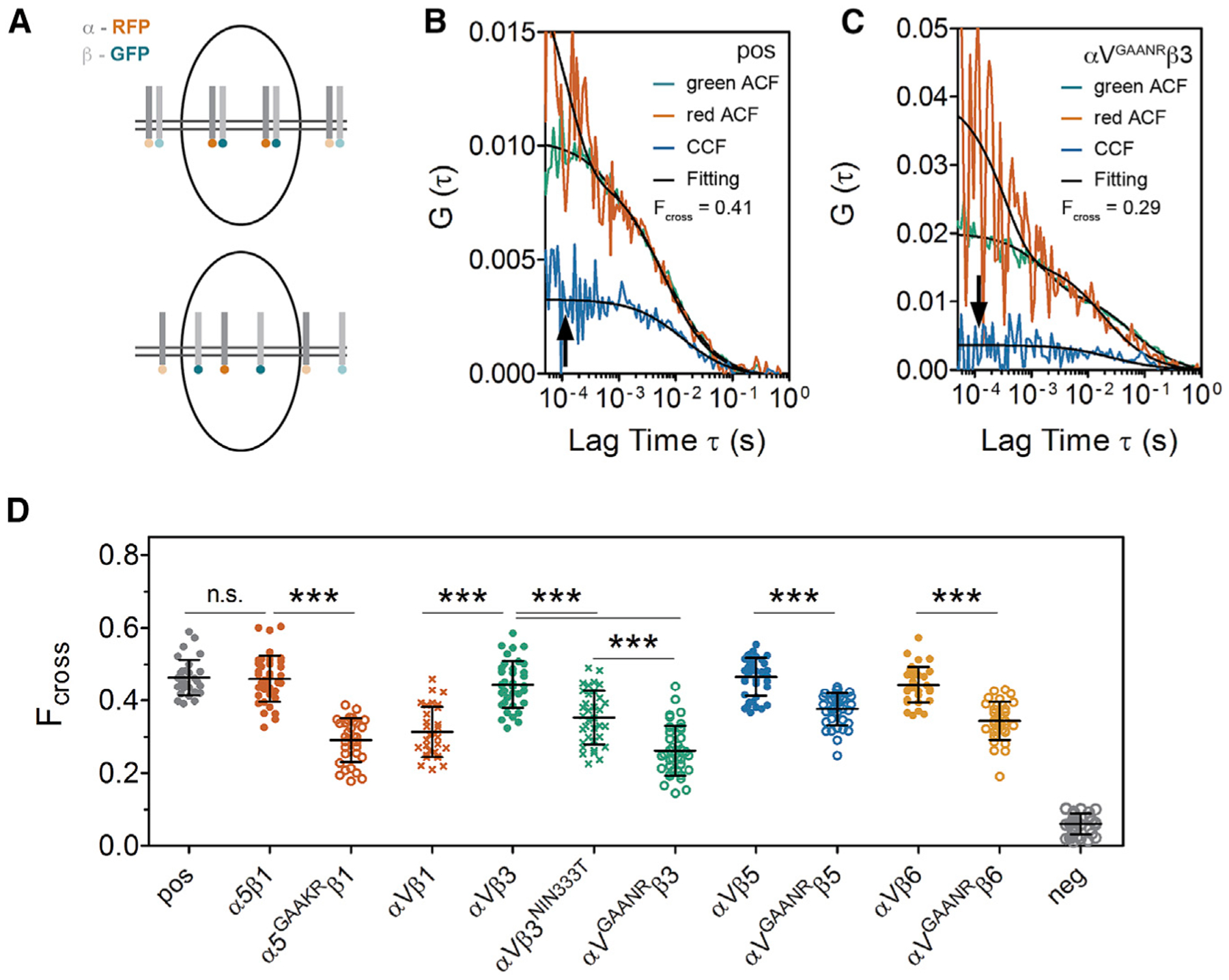
Integrin intra-heterodimer affinity inversely correlates with integrin activatability (A) Illustration of fluorescence cross-correlation spectroscopy (FCCS) measurements. The integrin α subunit cytoplasmic tail was tagged with RFP and the β subunit was tagged with GFP. When the two subunits move together through the confocal volume (upper panel), the green and red intensity fluctuations correlate, leading to a high cross-correlation curve (arrow in B); conversely, when the heterodimer subunits dissociate (lower panel), there is a lower cross-correlation curve (arrow in C). (B and C) FCCS measurements of the positive control (pos), which is a mem-GFP-RFP tandem fusion (B) and FCCS measure of αV^GAANR^β3 (C). The auto-correlation functions (ACFs) for each channel are shown in red and green while the cross-correlation between the two channels is in blue. Data fitting is shown in black. Measurements were performed on the cell surface in somite MCs (white cross in Figure 1E). (D) F_cross_ of different integrin heterodimers calculated from FCCS. A lower F_cross_ indicates a weaker intra-heterodimer association. pos, positive control, mem-GFP-RFP tandem; neg, negative control, co-expression of mem-GFP and mem-RFP. Data are mean ± SD. ***p < 0.0001, n.s., not significant (two-sided t test). See also Table 1 and Figure S3.

To measure intra-heterodimer affinity, we quantified the dissociation rate of different integrin heterodimers using FCCS (Jülich et al., 2015; Wang et al., 2016). In this study, we tagged the integrin α subunit cytoplasmic tail with RFP and the β with GFP and performed measurements on the cell surface of mesenchymal cells of live 10–13 somite stage embryos (white cross in Figure 1E). When the two subunits move together through the confocal volume, green and red intensity fluctuations correlate, leading to a high cross-correlation curve (Figures 4A and 4B). Conversely, heterodimer separation would result in a lower cross-correlation curve (Figures 4A and 4C). The strength of intra-heterodimer association was quantified using the fraction of molecules cross-correlating (F_cross_) and the apparent dissociation constant (K_D_). The positive control using mem-GFP-RFP and the negative control co-expressing mem-GFP and mem-RFP presented F_cross_ values of 0.46 and 0.06, respectively (Figure 4D; Table 1).

Integrin αVβ3 displayed F_cross_ and K_D_ values of 0.44 ± 0.07 and 134 ± 36 nM, respectively, both comparable to the positive control and suggesting a strong association between the two subunits (Figures 4D and S3; Table 1). In contrast, αVβ1, αVβ3^NIN333T^, and αV^GAANR^β3 showed significantly lower F_cross_ values of 0.26–0.35 (p < 0.0001) and higher apparent K_D_ values of 250–300 nM (p < 0.05 and p < 0.005). The activating mutation similarly reduced the intra-heterodimer affinities of the other integrin heterodimers; that is, α5β1 and α5^GAAKR^β1, αVβ5 and αV^GAANR^β5, and αVβ6 and αV^GAANR^β6 exhibited significant differences in F_cross_ ranging from 0.44–0.47 in the wild-type allele to 0.29–0.38 in activated alleles (p < 0.0001). The weaker associations of the activatable integrins are comparable to our previous measurements of adhesion between Cdh2 molecules expressed on adjacent cells in the presomitic mesoderm, which showed an F_cross_ of 0.21 ± 0.07 and a K_D_ of 200 ± 100 nM (Jülich et al., 2015). Although we expected α5β1 to have a weaker intra-heterodimer association than αVβ3, αVβ5, and αVβ6, we observed that these heterodimers were indistinguishable from the positive control in which the GFP and RFP are covalently bound. Thus, we are not able resolve the differences between these stronger interactions due to technical limitations. Overall, these results indicate that integrin αβ heterodimers associate tightly on the cell surface but have different dissociation rates. Notably, mutations that activate integrins reduce the association between heterodimer subunits and result in lower cell surface expression.

## DISCUSSION

In this study, we explore integrin activation biophysics *in vivo*. We find that integrins αVβ1 and α5β1 are activated along the somite boundary but that αVβ3, αVβ5, and αVβ6 are not activated. Accordingly, αVβ1 can partially compensate for the loss of integrin α5 during somitogenesis (Figure S2). Interestingly, we notice that αVβ1 and other activatable integrin alleles display poor cell surface expression unless they are stabilized by association with fibronectin along the somite boundary. FCCS measurements reveal that these activatable αV integrins have higher intra-heterodimer K_D_s and are thus are more likely dissociate on the cell membrane. In contrast, the stably expressed integrins such as αVβ3, αVβ5, and αVβ6 exhibit strong intra-heterodimer association and are never activated by the fibronectin matrix in this context. Our data indicate that integrin intra-heterodimer affinity determines how readily an integrin is activated.

Integrins equilibrate between three conformations: bent closed, extended closed, and extended open. Most cell surface integrins are in the bent-closed state. Only extended-open integrins have high ligand binding affinity, and cellular energy is required to stabilize this conformer (Li and Springer, 2018; Li et al., 2017; Zhu et al., 2013). Therefore, heterodimer instability reduces the energy barrier for this conformational change. Fibronectin not only drives integrin activation, but it also stabilized activated integrins on cell surface along the somite boundaries. This can be explained by the fact that integrins have fewer intra-heterodimer physical contacts in the extended conformation while fibronectin binding involves physical contact with both the α and β subunits (Campbell and Humphries, 2011). That activated integrins display poor cell surface expression in the mesenchyme may be explained by the slower recycling rate of active integrins (Arjonen et al., 2012).

Although integrin αVβ1 was first reported as the fibronectin receptor (Koivisto et al., 2000; Vogel et al., 1990; Zhang et al., 1993), much attention has been given to the role of αVβ3 in cellular adhesion to fibronectin. In the *in vivo* context studied here, β1 integrins are the most readily activated. One explanation is that β1 integrin is more heavily glycosylated than the other β subunits, which favors the active conformation, and it has been hypothesized that glycosylation levels may help establish different basal activities among integrin heterodimers (Li et al., 2017). Moreover, integrin β1 has the earliest embryonic lethality of any integrin mutant in mice (Hynes, 2002), suggesting that β1 integrins are tuned to activate in the mechanically soft environment of the early embryo. Notably, in zebrafish, integrin α5 is only required for embryogenesis and not adult viability, as injection of integrin α5 mRNA at the one-cell stage is sufficient to rescue the integrin α5 mutant to adulthood (Jülich et al., 2009).

Although αVβ3 is known as one of the primary fibronectin receptors, we did not observe integrin αVβ3 activation during zebrafish somitogenesis. Along with αVβ5 and αVβ6, αVβ3 is highly expressed on the cell membrane and exhibits a strong intra-heterodimer association. Even though these integrins are inactive, they still hold similar activation potential compared to α5β1 and αVβ1 when mutations weaken the intra-heterodimer association. It has been shown that increased intracellular tension can induce integrin αVβ3 activation and clustering in focal adhesions (Ballestrem et al., 2001; Cluzel et al., 2005). Thus, integrin αVβ3 is possibly tuned for a more mechanically rigid cellular environment later in development, such as in muscle or bone (Ablooglu et al., 2007; Sinanan et al., 2008), and/or tuned for a better ligand, such as vitronectin (Bachmann et al., 2020).

There are technical limits with FCCS such that we cannot distinguish the intra-heterodimer affinities of integrins α5β1, αVβ3, αVβ5, and αVβ6 and the covalent bond in our positive control. However, we suspect that the α5β1 heterodimer has a distinct molecular dynamics, for example, a faster conformational change, than these other heterodimers. Electron microscopy analysis of integrin extracellular domains found that most α5β1 heterodimers are in extended conformers whereas αVβ3 heterodimers are in the bent conformation (Miyazaki et al., 2018; Takagi et al., 2002). Integrin αVβ3 heterodimers might not only be more stable but also undergo less frequent conformational changes. Further exploration of this hypothesis requires single-molecule sensitive techniques with higher temporal resolution (Chen et al., 2017).

Integrins are unique in way that they transduce information across the cell membrane via an extensive conformational change. Our data implicate intra-heterodimer stability as a biophysical mechanism that determines how readily an integrin is activated. More broadly, our data suggest that these integrins operate in two distinct regimes. Integrins α5β1 and αVβ1 are highly activatable but provide a limited increase in cellular avidity (i.e., the number of binding sites a cell has for the ECM) because of low cell surface expression. In contrast, integrins αVβ3, αVβ5, and αVβ6 are not as readily activated but provide a larger potential increase in cellular avidity upon activation, due to their high cell surface expression, and therefore enable the cell to bear a greater mechanical load.

## STAR★METHODS

### RESOURCE AVAILABILITY

#### Lead contact

Further information and requests for resources and reagents should be directed to and will be fulfilled by the lead contact, Scott A. Holley (scott.holley@yale.edu).

#### Materials availability

All unique/stable reagents generated in this study are available from the lead contact without restriction.

#### Data and code availability

The mass spectrometry proteomics data have been deposited to the ProteomeXchange Consortium via the PRIDE (Perez-Riverol et al., 2019) partner repository with the dataset identifier PXD024665. The raw imaging and feature data have not been deposited in a public repository because of their size but are available from the corresponding authors upon request.

### EXPERIMENTAL MODEL AND SUBJECT DETAILS

#### Zebrafish care and strains

Zebrafish were maintained in accordance with standard protocols approved by the Institutional Animal Care and Use Committee at Yale University (IACUC). Wild-type strains used are TLF. The *MZα*5^−/−^ mutant line is a maternal zygotic mutant line using the *bfe*^*thl30*^ allele (Jülich et al., 2005). *cdh2*^−/−^ were generated by incrossing *cdh2*^+/−^ parents (Lele et al., 2002) and sorting progeny by phenotype. Fn^−/−^(*fn1a*^−/−^*; fn1b*^−/−^) were generated by incrossing *fn1a*^−/−^*; fn1b*^+/−^ parents and sorting progeny by phenotype (Guillon et al., 2020). The *hsp70:fn1a-mKIKGR* is a transgenic line with a heat-shock promoter driving expression of fibronectin 1a tagged with a photoconvertible protein mKikumeGR (Guillon et al., 2020). All experiments were performed on embryos within the first 25 hours of development prior to sex determination.

### METHOD DETAILS

#### Fluorescent protein constructs and *in vitro* transcription

All fluorophores were tagged at the Integrin C-terminus. The vector used was pCS2+. A spacer between Integrin and fluorophore was two amino acids. The spacer between two fluorophores in positive controls was seven amino acids. Integrin coding sequence were amplified via PCR from 16–25 hours post fertilization (hpf) cDNA generated from the TLF strain and cloned into pCS2+ vector. The PCR primers are listed in KEY RESOURCES TABLE. The particular GFP variant used was emeraldGFP, and RFP was tagRFP. Intracellular myristoylated membrane-anchored mem-GFP, mem-RFP, mem-GFP-RFP, Integrin α5-GFP, α5-RFP, and α5^FYLDD^-GFP constructs were previously described (Jülich et al., 2015). For the FRET-FLIM assay, the donor fluorophore Aquamarine (Aqm) was from pAquaN1 (Addgene, Plasmid #42888) and the acceptor fluorophore mCitrine (mCit) was kindly provided by Holger Knaut with an A207K mutation to make it monomeric. For co-immunoprecipitation experiments, C-terminal epitope FLAG (Trofka et al., 2012) tagged Integrin-GFP constructs were generated. New plasmids were made from PCR products of Integrin coding sequence, fluorophores, and double digestion products of pCS2+ vector from available constructs using Gibson Assembly Master Mix (NEB).

To generate a constitutively active Integrin αV^GAANR^, F1016A and F1017A mutations were generated via overlap extension PCR. Similarly, to introduce the glycan wedge to Integrin β3^NIN333T^, an NIN333T mutation was created. Amino acids D243A and D244A were changed in Integrin αV to create the RGD binding deficient Integrin αV^243AA^. For rescue experiments, the target sequence for the αV antisense morpholino was mutated in αV plasmids without altering the amino acid sequence.

To improve heterodimerization efficiency, we used a Venus YFP Bimolecular Fluorescence Complementation (BiFC) assay (Jülich et al., 2009). The amino terminal half of Venus (nV) is attached to Integrin α subunit and the carboxyl terminal half of Venus (cV) is attached to Integrin β subunit. Upon dimerization, the halves of Venus complement and fluoresce. pCS2+Hsα5-nV and pCS2+β1-cV from a previous study were used as templates for BiFC plasmid construction (Jülich et al., 2015).

For mRNA synthesis, the respective plasmids were linearized with NotI-HF (NEB), the mRNA *in vitro* transcribed with the Sp6 mMessage mMachine kit (Invitrogen), and cleaned with the Monarch DNA Cleanup kit (NEB). mRNA was injected into one-cell stage embryos.

#### Confocal microscopy

##### Sample preparation

Embryos at the 10–13 somite stage were manually dechorionated, embedded in 1% low-melt agarose (Bio-Rad) in a glass bottom dish with thickness of No. 1.5 (MatTek Corporation). The dorsal side of the embryo faces the cover glass. Experiments were performed at a room temperature (22°C).

##### Time-lapse

Acquisition of time-lapses was performed on a Zeiss LSM510 using a water immersion 40x objective (numerical aperture 1.2). Excitation was provided by the 488 nm laser line of an Argon ion laser and 543 nm laser line of HeNe laser. Laser power measured before the objective was 30 μW. Images were taken every 3 min to follow morphogenesis of the somite boundary cells. For each cell pair on the forming somite, line intensity along somite boundary (SB), anterior cell border (A), posterior cell border (P), and background measured in the nucleus (bg) were obtained using ImageJ. The intensity ratio was calculated as SB/A = (SB – bg)/(A – bg) and SB/p = (SB – bg)/(P – bg) every 6 min. Plots in Figure 1F are the average value with standard deviation from 15 cell pairs of 6 embryos.

##### FRET-FLIM

Fluorescence lifetime imaging microscopy was performed on a Zeiss LSM 880 Airyscan confocal microscope equipped with a Zeiss C-Apochromat 40x, numerical aperture 1.2, water immersion objective. Excitation was provided by a pulsed laser Ti:Sapphire laser (Mai Tai DeepSee, Spectra-Physics) with a repetition rate of 80 MHz at 820 nm. Laser power measured before the objective was 2 mW. A 460–500 nm fluorescence band-pass filter was used to detect the donor’s fluorescence. Images (256×100 pixels) were collected with pixel dwell time 65.9 μs and pixel size 0.69 μm, and summed 36 frames for region of interest (ROI) analysis or 100–120 frames (about 5 min) for pixel-to-pixel analysis. For images of the Venus BiFC, the excitation source was tuned to 960 nm and a 520–560 nm fluorescence band-pass filter was used. Images were acquired in the time-correlated single-photon counting (TCSPC) mode with resolution of 25 ps. Data acquisition and analysis were performed using the software SymPhoTime 64 (PicoQuant, version 2.1). Histograms of the photon arrival time of the ROI summing or for each pixel were analyzed by two-exponential reconvolution fits using the instrument response function (IRF). Data with peak maximum over 1000 counts were kept for analysis. Donor’s lifetime (τ_D_) was measured using the embryos expressing Integrin α5-Aqm, αV-Aqm, or mem-Aqm respectively. FRET efficiency (E_FRET_) was determined using the equation:

(1) $$E_{\text{FRET}}=1-\frac{\tau_{DA}}{\tau_{D}}$$

where τ_DA_ and τ_D_ are the lifetimes of the donor in the presence and absence of the acceptor, respectively. Lifetimes reported here are the amplitude-weighted mean fluorescence lifetimes.

To quantify the clustering state for each SB-MC pair measured, the intensity ratio after correction from intensity loss due to FRET was calculated as:

(2) $$SB/MC=\frac{I_{SB}/N_{SB}}{I_{MC}/N_{MC}}\times \frac{1-E_{\text{FRET},MC}}{1-E_{\text{FRET},SB}}$$

Where *I* is fluorescence intensity as the sum of photon counts in the ROI, N is the number of pixels of the ROI. Data reported are mean ± SD. Results of lifetime, E_FRET_, clustering quantification, and sample size are listed in Table S1.

For measurements in hsp70:fn1a-mKIKGR, embryos were heat-shocked for 30 minutes at 38°C (Guillon et al., 2020). To remove the green fluorescent signal from Fn1a-mKIKGR, photoconversion was performed on region of interest using a 405 nm laser (20 cycles, speed 9, z-scan cover the whole somite, laser power 500 μW, fully opened pinhole to minimize phototoxicity) before FRET-FLIM measurements.

#### Fluorescence cross-correlation spectroscopy (FCCS)

##### Theory

Fluorescence correlation spectroscopy (FCS) extracts information from fluorescence signal fluctuations as fluorophores pass through a small observation volume (around 1 femtoliter). This small observation volume is created by focusing a laser to a diffraction limited volume. Fluorescence fluctuations are generated by physical processes such as fluorophores moving in and out of the observation volume due to diffusion and flow. Fluctuations are also caused by processes which change the fluorescence property of the fluorophore during its residence time, such the photophysical and photochemical processes of fluorophore blinking and photobleaching. The fluorescence fluctuations are transformed by a temporal autocorrelation. The normalized autocorrelation function (ACF) can be written as:

(3) $$G(\tau )=\frac{F(t)F(t+\tau )}{F{\left(t\right)}^{2}}$$

where F(t) is the fluorescence intensity at time t, 〈〉 denotes time average, and t is the lag time.

In FCCS, two particle species are labeled with spectrally distinct fluorophores. Fluorescence signals from the two channels are cross correlated. When the two species bind to each other, they will move as a unit through the observation volume. This concurrent movement induces simultaneous fluctuations of the fluorescence signals in both channels and therefore produces an elevated cross-correlation function (CCF) curve. The normalized CCF is defined as,

(4) $$G(\tau )=\frac{F_{i}(t)F_{j}(t+\tau )}{F_{i}(t)F_{j}(t)}$$

where the subscripts i and j denote different fluorescent labels.

Experimental ACF and CCF curves are fitted with theoretical models. Assuming a Gaussian laser profile, the theoretical ACF for 3D free diffusion of one species with a triplet state is given by Aragon and Pecora (1976):

(5) $$G_{3D}(\tau )=\frac{1}{N}{\left(1+\frac{\tau }{\tau_{D}}\right)}^{-1}{\left[1+\frac{1}{K^{2}}\left(\frac{\tau }{\tau_{D}}\right)\right]}^{-1/2}f_{\text{trip}}(\tau )+G_{\infty }$$

in which,

(6) $$\tau_{D}=\frac{\omega_{0}^{2}}{4D}$$

(7) $$K=\frac{\omega_{z}}{\omega_{0}}$$

(8) $$f_{\text{trip}}(\tau )=\left(\frac{F_{\text{trip}}}{1-F_{\text{trip}}}\right)exp\left(-\frac{\tau }{\tau_{\text{trip}}}\right)+1$$

where N is the average number of molecules in the observation volume; τ_D_ is the diffusion time the fluorophore takes to pass through the observation volume; G_∞_ is the convergence value of the ACF for long times with the expected value of 0; D is diffusion coefficient; ω_0_ and ω_z_ are the radial and axial distances where the excitation intensity reaches 1/e^2^ of its value at the center of the observation volume, K describes the shape of the observation volume; F_trip_ is the fraction of the particles in the triplet state; τ_trip_ is the triplet state relaxation time. At higher laser intensities, a triplet state of the fluorophore can be induced. Typical triplet states have kinetics occurring on a timescale that is much faster than the diffusion time (Widengren et al., 1995; Widengren et al., 1999). Here, this equation describes intensity fluctuations generated from fluorophore blinking which is due to either the triplet state or due to isomerization.

For FCS measurements on the cell membrane, 2D or planar free diffusion models (Elson and Magde, 1974) are used:

(9) $$G_{2D,\;1p1t}(\tau )=\frac{1}{N}{\left(1+\frac{\tau }{\tau_{D}}\right)}^{-1}f_{\text{trip}}(\tau )+G_{\infty }$$

(10) $$G_{2D,\;1p}(\tau )=\frac{1}{N}{\left(1+\frac{\tau }{\tau_{D}}\right)}^{-1}+G_{\infty }$$

The Diffusion coefficient (D) can then be determined by:

(11) $$D=\frac{\tau_{D0}\times D_{0}}{\tau_{D}}$$

where τ_D0_ and D_0_ are diffusion time and diffusion coefficient of the calibration dye.

Assuming a 1:1 binding stoichiometry, the amplitude of the ACFs and CCF can then be expressed as a function of the count rate per particle per second (cps) and the concentrations of the particles involved (Hwang and Wohland, 2005; Liu et al., 2007):

(12) $$G_{g}(0)=\frac{{\left(\eta_{g}^{g}\right)}^{2}C_{g}+{\left(\eta_{r}^{g}\right)}^{2}C_{r}+{\left(q_{g}\eta_{g}^{g}+q_{r}\eta_{r}^{g}\right)}^{2}C_{gr}}{N_{A}V_{eff,\;g}{\left[\eta_{g}^{g}C_{g}+\eta_{r}^{g}C_{r}+\left(q_{g}\eta_{g}^{g}+q_{r}\eta_{r}^{g}\right)C_{gr}+\beta_{g}/\left(N_{A}V_{eff,\;g}\right)\right]}^{2}}$$

(13) $$G_{r}(0)=\frac{{\left(\eta_{g}^{r}\right)}^{2}C_{g}+{\left(\eta_{r}^{r}\right)}^{2}C_{r}+{\left(q_{g}\eta_{g}^{r}+q_{r}\eta_{r}^{r}\right)}^{2}C_{gr}}{N_{A}V_{eff,\;r}{\left[\eta_{g}^{r}C_{g}+\eta_{r}^{r}C_{r}+\left(q_{g}\eta_{g}^{r}+q_{r}\eta_{r}^{r}\right)C_{gr}+\beta_{r}/\left(N_{A}V_{eff,\;r}\right)\right]}^{2}}$$

(14) $$G_{x}(0)=\frac{\eta_{\text{g}}^{g}\eta_{g}^{r}C_{g}+\eta_{r}^{g}r_{r}^{r}C_{r}+\left(q_{g}\eta_{g}^{g}+q_{r}\eta_{r}^{g}\right)\left(q_{g}\eta_{g}^{r}+q_{r}\eta_{r}^{r}\right)C_{gr}}{N_{A}V_{eff,\;gr}\left[\eta_{g}^{g}C_{g}+\eta_{r}^{g}C_{r}+\left(q_{g}\eta_{g}^{g}+q_{r}\eta_{r}^{g}\right)C_{gr}+\beta_{g}/\left(N_{A}V_{eff,\;g}\right)\right]}\times {\left[\eta_{g}^{r}C_{g}+\eta_{r}^{r}C_{r}+\left(q_{g}\eta_{g}^{r}+q_{r}\eta_{r}^{r}\right)C_{gr}+\beta_{r}/\left(N_{A}V_{eff,\;r}\right)\right]}^{-1}$$

where G_g_(0) and G_r_(0) are the amplitudes of the ACF in the green (GFP) and red (RFP) channel, and G_x_(0) is the amplitude of the CCF; C_g_, C_r_, and C_gr_ are the concentrations of the free green, free red, and the complex particles, respectively; β_g_ and β_r_ are the uncor-related background count rate in the green and red channels; η is fluorophores cps calculated as:

(15) $$\eta =\frac{F_{\text{mean}}-\beta }{N_{cor}}$$

(16) $$N_{cor}=\frac{N_{app}\times {\left(F_{\text{mean}}-\beta \right)}^{2}}{F_{\text{mean}}^{2}}$$

where F_mean_ is the average fluorescence intensity, N_app_ is N, the number of particles obtained from fitting the correlation curve in Equation 5, 9, or 10, and N_cor_ is the background (b) corrected number of particles from N_app_ (Koppel, 1974). This correction is needed since background affects the measurement of the actual number of particles (Schwille et al., 1999). $\eta_{g}^{g}$ and $\eta_{r}^{g}$ are the cps of green- and red-labeled particles in the green channel; $\eta_{g}^{r}$ and $\eta_{r}^{r}$ are the cps of green- and red-labeled particles in the red channel; q_g_ and q_r_ are correction factors that account for changes in fluorescence yields upon binding via processes such as quenching or fluorescence energy transfer for the green and red particles; N_A_ is the Avogadro’s number; and V_eff_ is the effective observation volume calculated as:

(17) $$V_{eff,\;g}=\pi^{3/2}\omega_{0,g}{}^{2}\omega_{z,g}$$

(18) $$V_{eff,r}=\pi^{3/2}\omega_{0,r}{}^{2}\omega_{z,r}$$

(19) $$V_{eff,\;gr}={\left(\pi /2\right)}^{3/2}\left(\omega_{0,g}{}^{2}+\omega_{0,r}{}^{2}\right){\left(z_{0,g}{}^{2}+z_{0,r}{}^{2}\right)}^{1/2}$$

in which ω_0,g_, ω_0,r_, z_0,g_ and z_0,r_ can be experimentally obtained from calibration measurements using dyes with known diffusion coefficient using Equations 6 and 7. The diffusion time of the complex as obtained from G_gr_(τ) will be:

(20) $$\tau_{D,gr}=\frac{\omega_{0,g}{}^{2}+\omega_{0,r}{}^{2}}{8D}$$

Solving Equations 12–14 gives values of C_g_, C_r_, and C_gr_. To quantify the binding affinity, the dissociation constant K_D_ is defined as:

(21) $$K_{D}=\frac{C_{g}\times C_{r}}{C_{gr}}$$

Plotting the C_g_ × C_r_ against C_gr_, the slope of a linear fit yields the K_D_ ± fit error (Foo et al., 2012; Shi et al., 2009).

To estimate the binding or association strength qualitatively, normalized cross-correlation values, F_cross_, is defined as (Triffo et al., 2012):

(22) $$F_{\text{cross}}=\frac{G_{x}(0)}{\text{min}\left\{G_{r}(0),\;G_{g}(0)\right\}}$$

F_cross_ can range from 1 to 0, where 1 indicates perfect correlation or strongest interaction and 0 indicates no correlation or no interaction. In practice, an F_cross_ of 1 is not observed even in positive controls, due to photobleaching or different maturation efficiency of fluorophores, imperfect overlap of excitation and detection focal volumes, and energy transfer between the probes (Foo et al., 2012; Triffo et al., 2012). Also, F_cross_ of 0 is not observed because of the crosstalk of green fluorescence into the red channel.

##### Experiment

FCCS was performed on a Zeiss LSM 880 Airyscan confocal microscope equipped with a Zeiss C-Apochromat 40x, numerical aperture 1.2, water immersion objective. Image acquisition and measurement point selection were controlled by Zen Black software. Excitation was provided by the 488 nm laser line of an Argon ion laser and 561 nm laser line of HeNe laser. The laser power, measured before the objective, was 3 μW for 488 nm and 9 μW for 561 nm. This unequal power was selected to reduce the relative magnitude of green fluorescence bleed-through into the red channel (Jülich et al., 2015). The emitted light passed through a 34 μm pinhole and was separated by MBS 488/561/633 into two different detection ranges of 508–535 nm for the green channel and 606–668 nm for red channel set for internal 32-Channel GaAsP array. The correlator was set as 0.2 ms binning with 8 tau channels. The acquisition time for a measurement was 10 s.

##### Calibration

To quantify concentrations from FCCS measurements, parameters in Equations 12–14 need to be determined. The cps of GFP tagged particles was measured on mem-GFP and $\eta_{g}^{g}$ was determined using Equation 15 as 1096 ± 201 and the GFP cross talk $\eta_{g}^{r}$ was 3% of $\eta_{g}^{g}$ in red channels. Similarly, $\eta_{r}^{r}$ was determined as 194 ± 65 in experiments with only Integrin αV-RFP co-injected with unlabeled β3 and the RFP cross talk $\eta_{r}^{g}$ was 1% of $\eta_{r}^{r}$ in green channel. The individual cps is an average of at least 20 measurements from three embryos. Average background intensity in both channels is measured in embryos without microinjection and determined as 739 ± 165 and 857 ± 160 counts in the green and red channels. Our positive control of tandem mem-GFP-RFP showed noticeably lower $\eta_{g}^{g}$ and higher $\eta_{r}^{r}$ than GFP or RFP alone. We attribute this to fluorescence energy transfer and hence correction factors of q_g_ = 0.5 and q_r_ = 1.5 were used for the positive control. In Integrin α5β1 and αVβ3 datasets, a majority of measurements (> 70%) displayed a similar phenomenon but was less marked than the positive control. The correction factors were determined as q_g_ = 0.7, q_r_ = 1.3 for Integrin α5β1 and q_g_ = 0.8, q_r_ = 1.2 for Integrin αVβ3, αVβ5, and αVβ6. In other binding experiments, less than 20% measurements showed such changes and most changes were less than 10% in cps and hence q_g_ and q_r_ = 1 were used. V_eff_ was determined by Equations 17–19 using Atto 488 (Sigma) (D = 400 μm^2^s^−1^)(Kapusta, 2010) and Atto 565 (Sigma)(D = 392 μm^2^s^−1^) (Braun et al., 2012). A droplet of 60 μL of 5 nM sample solution (in 1x PBS) was used. Laser power before the objective was 25 μW. Veff,g, Veff,r, Veff,gr were determined to be 9.38×10^−16^ L, 8.26×10^−16^ L, and 8.82×10^−16^ L, respectively. Diffusion times of Atto 488 and Atto 565 were estimated as 53 ± 2 ns and 51 ± 4 ns and used to calculate diffusion coefficients using Equation 11. Diffusion coefficients of complexes detected in CCF were calculated using Equation 20. Note that the majority measurements of Integrin α^GAAXR^β and negative control cannot be solved using Equations 12–14. Thus, we solved these equations by ignoring cross talk between green and red channel ($\eta_{r}^{g}$, $\eta_{r}^{g}=0$) and the background (β_g_, β_r_ = 0). Using this simplification to treat αVβ1 and αVβ3^NIN333T^ datasets yield significantly smaller K_D_. Therefore, the K_D_ of α^GAAXR^β is likely underestimated, i.e., the affinity is likely weaker.

##### Data fitting

Data were fit using QuickFit 3.0 (https://github.com/jkriege2/QuickFit3/releases/tag/GIT4464%2F4465) with the Levenberg-Marquardt algorithm. Measurement curves of dyes in solution were fit with 3D-normal diffusion of one diffusion component with a triplet (Equation 5). For measurements on the cell membrane, ACF curves were fit with 2D-normal diffusion of one diffusion component with a triplet (Equation 9) and CCF curves were fit with a diffusion-only model (Equation 10). Measurements yielding greater than approximately 150 molecules per observation volume were discarded. Datasets with a number of particle ratio (N_cor,G_/N_cor,R_) between 0.5 to 2 were kept to avoid a biased estimation of interaction (Foo et al., 2012; Shi et al., 2009). Linear regression for K_D_ measurements and their statistics were performed using GraphPad Prism (GraphPad Software).

#### Mass spectrometry (MS)

##### Sample preparation

For each replicate, 120 embryos were injected with mRNA (250 ng/μL, 450 pg) encoding GFP-FLAG-tagged Integrins at the one cell stage, raised to 10–13 somite stage, and then dechorionated using pronase (Sigma). After rinsing with modified Ringer’s solution (116mM NaCl, 3mM KCl, 4mM CaCl_2_, 1mM MgCl_2_, 5mM HEPES pH 7.8), embryos were incubated in modified Ringer’s solution containing 5mM DTBP (Dimethyl-3,3′-Dithiobispropionimidate, Thermo Scientific) at 28.6°C for 5 hr. Then, the crosslinking reaction was quenched by incubating in modified Ringer’s solution containing 50mM Tris-HCl pH 7.6 on ice for 20 min. Embryos were then transferred into 0.2 to 0.4 mL lysis buffer (50mM Tris pH7.6, 150mM NaCl, 1mM EDTA, 10% glycerol, one tablet cOmplete protease inhibitor cocktail, 5% Triton X-100, 0.1% IGEPAL), disrupted manually in Eppendorf tubes with a pestle (Fisherbrand), incubated on ice 30 min with gentle vortexing every 5 min, and clarified by 10 min centrifugation at 10,000 × g. The supernatant was transferred to fresh tubes and kept on ice before immunoprecipitation.

##### Immunoprecipitation (IP)

The anti-FLAG M2 affinity gel (A2220, Sigma) was prepared according to the manufacturer’s instructions. Briefly, 20 μL packed gel per sample was prepared by washing three times briefly in 400 μL TBS, once for 5 min in 500 μL 0.1M glycine pH 3.5, four times in 400 μL TBS. All centrifugation was at 7,000 × g except last two wash steps which are at 10,000 × g. Samples were exposed to affinity gel overnight at 4°C with gentle agitation. Immunoprecipitates (IPs) were washed four times in 500 μL TBS. After washes, 35 μL 2x Laemmli Sample Buffer (Bio-Rad) was added to affinity resins, and the mixture was incubated at 95°C for 7 min, followed by incubation on ice for 1 min and centrifugation for 30 s at 8,200 × g. The supernatant (about 30 μL) was transferred to a fresh tube and kept at 4°C or −20°C (for longer storage) until running on 10% sodium dodecyl sulfate–polyacrylamide gel (SDS-PAGE).

##### Coomassie staining

Following SDS-PAGE, total protein was visualized by incubating gels in Coomassie staining solution (0.1% (w/v) Coomassie Brilliant Blue G 250 (AmericanBio), 10% (v/v) Acetic Acid, 45% (v/v) Methanol) for 2 hr at room temperature. Gels were then destained in detaining buffer (10% (v/v) Acetic Acid, 20% (v/v) Methanol). Between each step, the gel was washed with excess distilled H_2_O. After destaining, lanes were sliced into 2 slices, higher than 75 kDa and 25 – 75 kDa. Samples were kept at −20°C before being sent for MS analysis.

##### In-gel proteolytic digestion

Gel slices were cut into small pieces and washed for 10 min with water, followed by washing for 30 min with 1 mL 50% acetonitrile (ACN)/100 mM NH_4_HCO_3_ (ammonium bicarbonate, ABC). The samples were reduced by the addition of 80 μL 4.5 mM dithiothreitol (DTT) in 100 mM ABC with incubation at 37°C for 30 minutes. The DTT solution was removed and the samples were cooled to room temperature. The samples were alkylated by the addition of 80 μL 10mM iodoacetamide (IAN) in 100mM ABC with incubation at room temperature in the dark for 30 minutes. The IAN solution was removed and the gels were washed for 15 minutes with 900 μL 50% ACN/100 mM ABC, then washed for 15 minutes with 900 μL 50% ACN/25 mM ABC. The gels were briefly dried by SpeedVac, then resuspended in 80 μL of 25mM ABC containing 400 ng of digestion grade trypsin (Promega, V5111) and incubated at 37°C for 16 hours. The supernatant containing tryptic peptides was transferred to a new Eppendorf tube, and the gel band was extracted with 350 μL of 80% acetonitrile/0.1% trifluoroacetic acid (TFA) for 15 minutes. Supernatants were combined and dried by speed vacuum. Peptides were dissolved in 25 μL MS loading buffer (2% ACN, 0.2% TFA), with 5 μL injected for LC-MS/MS analysis.

##### LC-MS/MS analysis

LC-MS/MS analysis was performed on a Thermo Scientific Q Exactive Plus equipped with a Waters nanoAcquity UPLC system utilizing a binary solvent system (A: 100% water, 0.1% formic acid; B: 100% acetonitrile, 0.1% formic acid). Trapping was performed at 5 μL/min, 99.5% Buffer A for 3 min using a Waters ACQUITY UPLC M-Class Symmetry C18 Trap Column (100Å, 5 μm, 180 μm × 20 mm, 2G, V/M). Peptides were separated at 37°C using a Waters ACQUITY UPLC M-Class Peptide BEH C18 Column (130Å, 1.7 μm, 75 μm × 250 mm) and eluted at 300 nL/min with the following gradient: 3% buffer B at initial conditions; 5% B at 2 minutes; 25% B at 140 minutes; 40% B at 165 minutes; 90% B at 170 minutes; 90% B at 180 min; return to initial conditions at 182 minutes. MS was acquired in profile mode over the 300–1,700 m/z range using 1 microscan, 70,000 resolution, AGC target of 3E6, and a maximum injection time of 45 ms. Data dependent MS/MS were acquired in centroid mode on the top 20 precursors per MS scan using 1 microscan, 17,500 resolution, AGC target of 1E5, maximum injection time of 100 ms, and an isolation window of 1.7 m/z. Precursors were fragmented by HCD activation with a collision energy of 28%. MS/MS were collected on species with an intensity threshold of 1E4, charge states 2–6, and peptide match preferred. Dynamic exclusion was set to 20 s.

##### Peptide and protein identification

Tandem mass spectra were extracted by Proteome Discoverer software (version 2.2.0.388, Thermo Scientific) and searched in-house using the Mascot algorithm (version 2.6.1, Matrix Science). The data were searched against a Uniprot reference proteome for *Danio rerio* (46,927 sequences). Search parameters included trypsin digestion with up to 2 missed cleavages, peptide mass tolerance of 10 ppm, and MS/MS fragment tolerance of 0.02 Da. Cysteine carbamidomethylation and methionine oxidation were configured as variable modifications. Normal and decoy database searches were run, with the confidence level was set to 95% (p < 0.05). Scaffold (version Scaffold_4.9.0, Proteome Software Inc., Portland, OR) was used to validate MS/MS based peptide and protein identifications. Peptide identifications were accepted if they could be established at greater than 95.0% probability by the Scaffold Local FDR algorithm. Protein identifications were accepted if they could be established at greater than 99.0% probability and contained at least 2 identified peptides. Protein probabilities were assigned by the Protein Prophet algorithm (Nesvizhskii et al., 2003). Proteins that contained similar peptides and could not be differentiated based on MS/MS analysis alone were grouped to satisfy the principles of parsimony. Proteins sharing significant peptide evidence were grouped into clusters. The cluster representative was used for further quantification. The two pieces of each biological sample were analyzed separately by MS and results were combined in Scaffold for further quantification.

##### Quantification

Label-free quantification of relative protein abundance was performed using intensity-based absolute quantification (iBAQ) (Hogl et al., 2013; Schwanhäusser et al., 2011). The iBAQ is the sum of all the peptides intensities divided by the number of observable peptides of a protein and was calculated on the basis of the unweighted spectral count assigned to each identified protein by Scaffold. To normalize the data, median normalized iBAQ (miBAQ) was calculated for each sample. Full data are listed in Table S2.

##### Hierarchical clustering analysis

Hierarchical clustering analysis was performed in RStudio. For each dataset, mean miBAQ was calculated for proteins with presence in at least two replicates. Note that if there were only two non-zero values, the mean was the average of the two. Median normalized mean miBAQ were used for hierarchical clustering on the basis of Euclidean distances and complete linkage matrix. Clustering results were visualized using the pheatmap package (version 1.0.12).

#### Morpholino injection and rescue experiments

The Integrin αV antisense morpholino (αV^MO^) was αV1 described by Ablooglu et al. (2010). The morpholino (MO) was obtained from Gene Tools and injected into the yolk of one-cell stage embryos a concentration of 250 μM, approximately 3.8 ng per embryo. For rescue experiments, 250 ng/μL mRNA, approximately 450 pg was co-injected with MO into *MZα*5^−/−^ mutant embryos. Integrins injected were Aquamarine or mCitrine tagged as in FRET-FLIM experiments. Untagged proteins yielded similar results (data not shown). *In situ* hybridization for *xirp2a* (ZFIN: ZDB-PUB-010810–1; https://zfin.org/ZDB-PUB-010810-1) using Dig-labeled antisense probes and NBT/BCIP staining followed standard protocols.

### QUANTIFICATION AND STATISTICAL ANALYSIS

Statistical details of experiments can be found in the figure legends. Results are reported as mean ± standard deviation. Comparisons were performed using unpaired two-tailed t test. Comparisons between K_D_ linear fits were performed using analysis of covariance (ANCOVA). All statistical analysis was performed using GraphPad Prism (GraphPad Software).

## Supplementary Material

1

Table S3

## Figures and Tables

**Table 1.** Summary of FCCS measurements

Measurement	F_cross_	K_D_ (nM)	D (μm^2^/s) green	D (μm^2^/s) red	D (μm^2^/s) cross	Total cell nos.	Total embryo nos.
pos	0.46 ± 0.05	114 ± 21	1.97 ± 0.74	1.78 ± 0.71	1.75 ± 1	30	6
α5β1	0.46 ± 0.06	100 ± 32	0.63 ± 0.29	0.78 ± 0.28	0.64 ± 0.33	40	5
α5^GAAKR^β1	0.29 ± 0.06	238 ± 53	0.88 ± 0.51	0.92 ± 0.38	1.03 ± 0.7	31	5
αVβ1	0.31 ± 0.07	277 ± 58	0.81 ± 0.35	0.87 ± 0.36	0.7 ± 0.62	30	7
αVβ3	0.44 ± 0.07	134 ± 36	0.59 ± 0.26	0.72 ± 0.33	0.7 ± 0.45	36	5
αVβ3^NIN333T^	0.35 ± 0.07	250 ± 46	1.16 ± 0.68	0.84 ± 0.41	0.93 ± 0.63	35	6
αV^GAANR^β3	0.26 ± 0.07	300 ± 54	1.07 ± 0.53	1.18 ± 0.53	1.01 ± 0.87	38	7
αVβ5	0.47 ± 0.05	95 ± 20	0.8 ± 0.29	0.75 ± 0.27	0.63 ± 0.34	39	6
αV^GAANR^β5	0.38 ± 0.04	198 ± 28	0.69 ± 0.31	0.77 ± 0.26	0.88 ± 0.59	37	5
αVβ6	0.44 ± 0.05	119 ± 17	0.68 ± 0.18	0.73 ± 0.34	0.61 ± 0.39	34	6
αV^GAANR^β6	0.34 ± 0.05	222 ± 29	0.84 ± 0.41	0.82 ± 0.49	0.83 ± 0.73	34	7
neg	0.06 ± 0.03	–	2.6 ± 1.16	2.46 ± 0.97	–	40	6

Data are mean ± SD, except K_D_ = slope ± fit error. Note that differences in K_D_ of α5β1, αVβ3, αVβ5, αVβ6, and pos are not statistically significant. See K_D_ plot and statistics in Figure S3. K_D_, dissociation constant; D, diffusion coefficient. pos, positive control (mem-GFP-RFP tandem); neg, negative control (co-expression of mem-GFP and mem-RFP).

**Table:** KEY RESOURCES TABLE

REAGENT or RESOURCE	SOURCE	IDENTIFIER
Chemicals, peptides, and recombinant proteins		
Pronase	Sigma-Aldrich	Cat# 10165921001
DTBP (Dimethyl-3,3’-Dithiobispropionimidate)	Thermo Scientific	Cat# PI20665
cOmplete protease inhibitor cocktail	Sigma-Aldrich	Cat# 11697498001
anti-FLAG M2 affinity gel	Sigma-Aldrich	Cat# A2220
2x Laemmli sample buffer	Bio-Rad	Cat# 1610737
Coomassie brilliant blue G 250	AmericanBio	Cat# 6104-58-1
20% SDS solution	AmericanBio	Cat# AB01922–00500
Triton X-100	AmericanBio	Cat# AB02025–00500
IGEPAL® CA-630	Sigma-Aldrich	Cat# I3021
Deposited data		
Proteomics data	PRIDE	PXD024665
Experimental models: organisms/strains		
Zebrafish (Danio rerio), TLF strain	ZIRC	RRID:ZIRC_ZL86
strain *cdh2* mutant *tm101*	(Lele et al., 2002)	RRID: ZFIN_ZDB-GENO-080110–3
strain *MZ itga5* mutant *thl30*	(Jülich et al., 2005)	ZIRC: ZL2023
*fn1a; fn1b* double mutant	(Guillon et al., 2020)	N/A
Tg(*hsp70:fn1a-mKIKGR*)	(Guillon et al., 2020)	N/A
Oligonucleotides		
Forward primer for itgαV coding sequence amplification from cDNA: ATGGGCAAACACTTCGTCCGC	Eurofins Genomics LLC	N/A
Reverse primer for itgαV coding sequence amplification from cDNA: GGCTTCAGTGTTTCGGTCTCC	Eurofins Genomics LLC	N/A
Forward primer for Itgβ3 coding sequence amplification from cDNA: ATGGAGGAAACTTCAGCCAAA	Eurofins Genomics LLC	N/A
Reverse primer for Itgβ3 coding sequence amplification from cDNA: GTCTTTGCCTCGATATGTGAT	Eurofins Genomics LLC	N/A
Forward primer for Itgβ5 coding sequence amplification from cDNA: ATGTGGAAACTTTGCTCATCTAC	Eurofins Genomics LLC	N/A
Reverse primer for Itgβ5 coding sequence amplification from cDNA: GTGGACTCCTCCGTTCAGTGAC	Eurofins Genomics LLC	N/A
Forward primer for Itgβ6 coding sequence amplification from cDNA: ATGGGGATTGTTTCACTCTGC	Eurofins Genomics LLC	N/A
Reverse primer for Itgβ6 coding sequence amplification from cDNA: GCGGCCTAAAGAAACATCACT	Eurofins Genomics LLC	N/A
Forward primer for Itgβ8 coding sequence amplification from cDNA: ATGCAGGACAACCTGGATCGG	Eurofins Genomics LLC	N/A
Reverse primer for Itgβ8 coding sequence amplification from cDNA: CCAGGCGTCCCCGATGGGCAT	Eurofins Genomics LLC	N/A
Integrin αV antisense morpholino: Integrin_αV1: AGTGTTTGCCCATGTTTTGAGTCTC	Gene Tools, LLC	N/A
Software and algorithms		
SymPhoTime 64	PicoQuant	version 2.1
QuickFit 3.0	https://github.com/jkriege2/QuickFit3/releases/tag/GIT4464%2F4465	N/A
GraphPad Prism	GraphPad Software	N/A
Scaffold	Proteome Software Inc., Portland, OR	version Scaffold_4.9.0
R (RStudio)	(R Core Team) https://www.rstudio.com/	N/A

## Highlights

- Integrin biophysics is quantified in live zebrafish embryos
- Integrin conformational changes are assayed using FRET-FLIM
- Intra-heterodimer affinities are measured via FCCS
- Activatable heterodimers have weak affinities and low cell surface expression.
