# Supplementary material for: Integrin intra-heterodimer affinity inversely correlates with integrin activatability
Source: Cell Rep. Author manuscript; Available in PMC 2021 Jun 25. (PMC8227800; doi:10.1016/j.celrep.2021.109230)
Supplement: 1 [file NIHMS1713048-supplement-1.pdf]

Cell Reports, Volume *35*

Supplemental information

Integrin intra-heterodimer affinity inversely correlates with integrin activatability

Guangyu Sun, Emilie Guillon, and Scott A. Holley

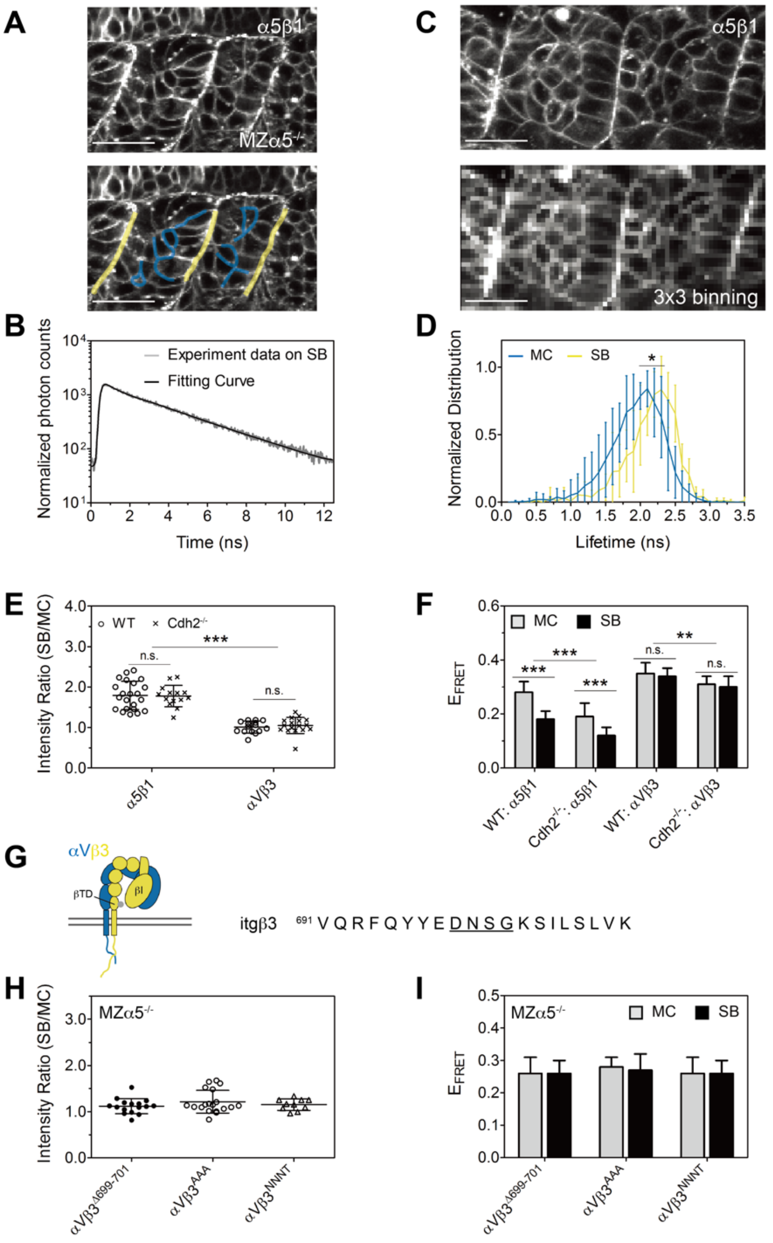

Figure S1. Region of interest selection for clustering and fluorescent lifetime experiments, and Cdh2 repression and the deadbolt model do not account for lack of $\alpha V\beta 3$ activation. Related to Figures 1 and 2.

(A) Top panel: two photon excitation imaging of $\alpha 5$-Aqm co-expressed with $\beta 1$-mCit as in Figure 1G. Lower panel: regions of interest used for pixel binning are indicated in blue for somite mesenchymal cells (MC) and yellow for somite boundary (SB). Scale bars, 30 μm.

(B) Normalized photon counts histogram of pixels in SB from the image in A. Experimental data: grey; fitting curve: black. Fluorescence lifetime τ is the time at which the intensity has decayed to 1/e.

(C) Top panel: raw two photon excitation imaging of $\alpha 5$-Aqm co-expressed with $\beta 1$-mCit as in Figure 2B. Lower panel: 3x3 pixel binning is used for lifetime analysis in each pixel.

(D) Normalized distribution of amplitude-weighted mean fluorescence lifetime from pixels in the SB and MC. Data are mean ± SD from n = 6 measurements in 4 WT embryos; *$p < 0.05$, two-sided t-test.

(E, F) Clustering quantification (E) and FRET efficiency (E_{FRET})(F) of Integrin $\alpha 5\beta 1$ and $\alpha V\beta 3$ in wild type (WT) and *cdh2*$^{-/-}$ mutant embryos. Integrin $\alpha 5\beta 1$: WT, n = 22 measurements (8 embryos); *cdh2*$^{-/-}$, n = 14 (12). Integrin $\alpha V\beta 3$: WT, n = 15 (8); *cdh2*$^{-/-}$ mutant, n = 17 (7). Data are mean ± SD, ***$p < 0.0001$, **$p < 0.01$, n.s., not significant, two-sided t-test.

(G) Integrin $\alpha V\beta 3$ in inactive conformation. In deadbolt model, association between β tail domain (βTD) and βI head in Integrin $\beta 3$ lock $\alpha V\beta 3$ in an inactive state structure. This association are mediated through the CD-loop (grey dot/underlined) within the βTD domain (partly shown).

(H, I) Clustering quantification (H) and FRET efficiency (E_{FRET})(I) (H) Clustering of $\alpha V\beta 3$ alleles in MZ$\alpha 5^{-/-}$ embryos. $\alpha V\beta 3^{\Delta 699\text{-}701}$, with $\beta 3^{699\text{-}701}$ deleted, n = 16 (14); $\alpha V\beta 3^{AAA}$, residues $\beta 3^{699\text{-}701}$ changed to AAA, n = 19 (13); $\alpha V\beta 3^{NNNT}$, changed $\beta 3^{699\text{-}702}$ to NNNT to induce a glycan wedge, n = 10 (7). Data are mean ± SD.

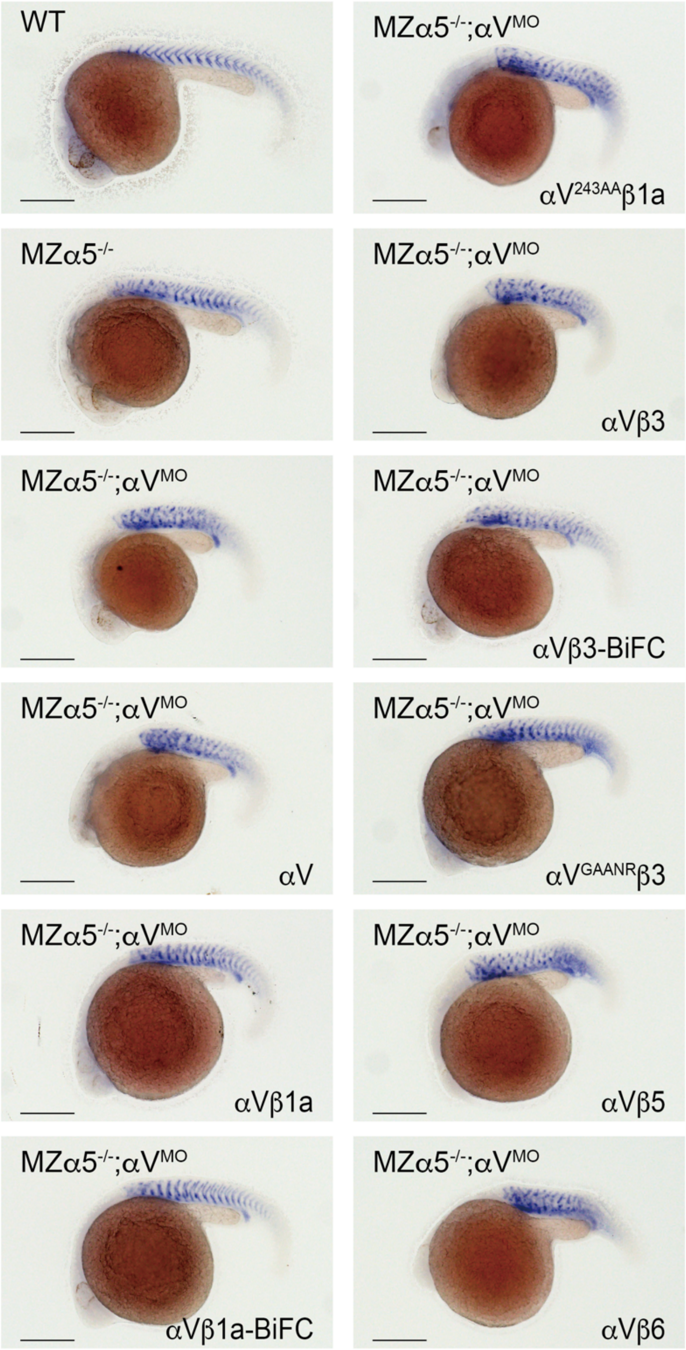

$MZ\alpha5^{-/-};\alpha V^{MO}$	# of Embryos with rescued posterior SB	# of Experiments
-	0/133	5
αV	0/128	3
$\alpha V\beta1a$	87/161	3
$\alpha V\beta1a$-BiFC	87/120	4
$\alpha V\beta1b$	57/118	3
$\alpha V^{243AA}\beta1a$	7/152	3
$\alpha V\beta3$	0/129	3
$\alpha V\beta3$-BiFC	8/158	4
$\alpha V^{GAANR}\beta3$	91/133	3
$\alpha V\beta5$	2/134	3
$\alpha V\beta6$	11/151	3

Figure S2. Integrin $\alpha V\beta1$ functions semi-redundantly with $\alpha5\beta1$ in zebrafish somite boundary formation. Related to Figure 3.

In situ hybridization for *xirp2a* to highlight somite boundaries at 28 hpf in αV antisense morpholino-injected (αV^{MO}) $MZ\alpha5^{-/-}$ zebrafish embryos. $MZ\alpha5^{-/-}$ embryos have defects in the anterior ~8 somites, but the posterior somites are normal. Knockdown of αV in $MZ\alpha5^{-/-}$ embryos leads to defects in both the anterior and posterior somites. mRNAs for different integrin heterodimers (indicated in the lower right corner) were co-injected to assay for rescue of posterior somite boundaries. Scale bars, 100 μm. Rescue experiments results are listed in the table. Neither $\alpha V\beta3$ nor RGD binding deficient $\alpha V^{243AA}\beta1a$ rescue, but activated $\alpha V^{GAANR}\beta3$, $\alpha V\beta1a$, $\alpha V\beta1b$ and $\alpha V\beta1a$-BiFC do rescue the formation of the posterior somite boundaries.

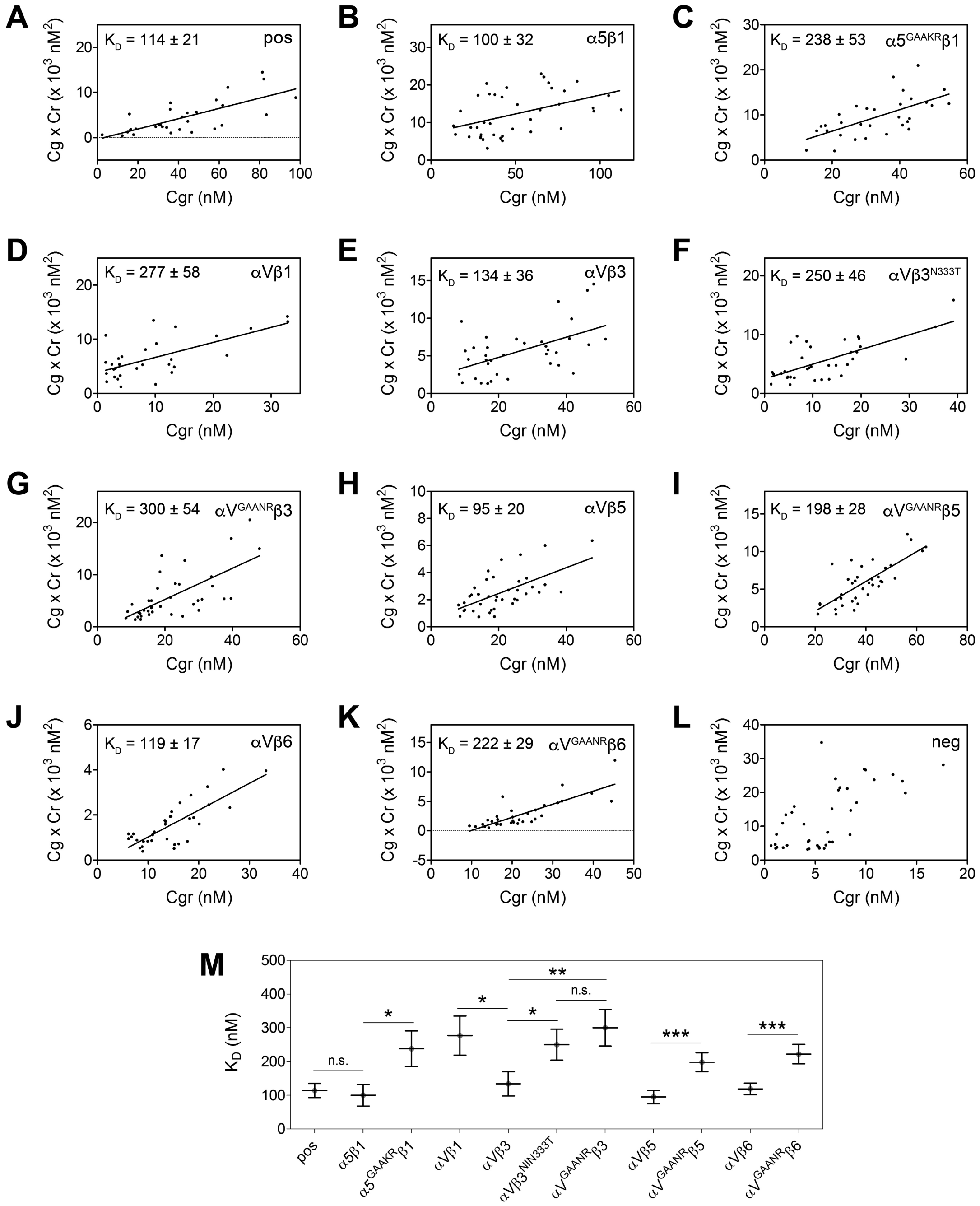

Figure S3. Apparent dissociation constant, K_D, of different Integrin heterodimers, related to Figure 4.

(A-L) Scatter plots of the product of the concentrations of free green and free red particles (C_g x C_r) versus the concentration of green – red complexes (C_{gr}). The K_D is calculated as the slope of the linear least-squares fit and indicated in the upper left corner. Data are slope ± fitting error. pos, positive control, membrane-GFP-RFP tandem fusion protein; neg, negative control, co-expression of membrane-GFP and membrane-RFP. Sample size listed in Table 1.

(M) Statistical tests of the slopes, ***$p < 0.0001$, **$p < 0.005$ *$p < 0.05$, n.s., not significant, analysis of covariance (ANCOVA). Data are slope ± fitting error. Note that K_D's of pos, α5β1, αVβ3, αVβ5, αVβ6 are not significantly different.

Table S1. A Summary of Fluorescence Lifetime and FRET Efficiency (E_{FRET}) Measurements, related to Figures 1-3.

Measurements	Lifetime (ns)		E_{FRET}		Cluster (SB/MC)	SB/MC pair No.	Total Embryo No.	Figures
	MC	SB	MC	SB				
WT:α5	2.83 ± 0.08	2.86 ± 0.08	-	-	-	16	7	τ_D for α5 heterodimer
WT:αV*	2.83 ± 0.07	2.87 ± 0.06	-	-	-	16	8	τ_D for αV heterodimer
WT:mem-Aqm	2.91 ± 0.11	2.92 ± 0.10	-	-	-	15	9	τ_D for neg
WT:α5/α5	2.65 ± 0.12	2.64 ± 0.12	0.06 ± 0.04	0.08 ± 0.04	-	17	9	Inter-heterodimer FRET test
WT:αV/αV*	2.55 ± 0.12	2.57 ± 0.13	0.10 ± 0.04	0.11 ± 0.05		15	9	Inter-heterodimer FRET test
WT:pos	1.29 ± 0.08	1.37 ± 0.08	0.54 ± 0.03	0.52 ± 0.03	-	18	9	Fig. 1M, 2C
WT:neg	2.80 ± 0.09	2.82 ± 0.07	0.04 ± 0.03	0.04 ± 0.02	-	15	7	Fig. 1M, 2C
MZα5$^{-/-}$:α5β1	2.15 ± 0.14	2.38 ± 0.11	0.24 ± 0.05	0.17 ± 0.04	1.86 ± 0.39	18	12	Fig. 1M, 2C
MZα5$^{-/-}$:αVβ1	-	2.40 ± 0.10	-	0.16 ± 0.03	1.86 ± 0.37	20 (21)	12 (13)	Fig. 1M, 2C
MZα5$^{-/-}$:αVβ3	1.98 ± 0.09	2.03 ± 0.10	0.30 ± 0.03	0.29 ± 0.03	1.11 ± 0.24	18	8	Fig. 1M, 2C
MZα5$^{-/-}$:αVβ5	2.01 ± 0.11	2.08 ± 0.09	0.29 ± 0.04	0.28 ± 0.03	1.11 ± 0.17	16	14	Fig. 1M, 2C
MZα5$^{-/-}$:αVβ6	1.89 ± 0.11	1.91 ± 0.07	0.33 ± 0.04	0.33 ± 0.02	1.02 ± 0.17	19	9	Fig. 1M, 2C
MZα5$^{-/-}$:α5^{GAAKR}β1	-	2.63 ± 0.17	-	0.08 ± 0.06	-	18	9	Fig. 2L, M
MZα5$^{-/-}$:αV^{GAANR}β3	-	2.38 ± 0.18	-	0.17 ± 0.06	2.07 ± 0.51	14 (16)	9 (9)	Fig. 2L, M
MZα5$^{-/-}$:αVβ3^{NIN333T}	2.14 ± 0.11	2.36 ± 0.12	0.25 ± 0.04	0.18 ± 0.04	1.63 ± 0.32	21	15	Fig. 2L, M
MZα5$^{-/-}$:αV^{GAANR}β5	1.91 ± 0.11	2.26 ± 0.13	0.32 ± 0.04	0.21 ± 0.04	1.62 ± 0.26	15	8	Fig. 2L, M
MZα5$^{-/-}$:αV^{GAANR}β6	-	2.47 ± 0.13	-	0.14 ± 0.05	2.03 ± 0.43	15(17)	7(8)	Fig. 2L, M
Fn$^{-/-}$:α5β1	2.10 ± 0.09	2.14 ± 0.08	0.26 ± 0.03	0.25 ± 0.03	1.13 ± 0.20	20	9	Fig. 3H, I
Fn$^{-/-}$:αVβ1-BiFC	-	-	-	-	1.21 ± 0.23	19	11	Fig. 3H, I
MZα5$^{-/-}$:α5^{FYLDD}β1	2.26 ± 0.12	2.31 ± 0.14	0.20 ± 0.04	0.19 ± 0.05	1.12 ± 0.19	16	8	Fig. 3H, I
hsp70:fn1a;αVβ3	1.98 ± 0.15	1.99 ± 0.15	0.30 ± 0.05	0.31 ± 0.05	1.23 ± 0.23	15	7	Fig. 3H, I
WT:α5β1	2.04 ± 0.12	2.34 ± 0.09	0.28 ± 0.04	0.18 ± 0.03	1.79 ± 0.35	22	8	Fig. S1E, F
Cdh2$^{-/-}$:α5β1	2.29 ± 0.13	2.52 ± 0.10	0.19 ± 0.05	0.12 ± 0.03	1.78 ± 0.27	14	12	Fig. S1E, F
WT:αVβ3	1.85 ± 0.11	1.89 ± 0.10	0.35 ± 0.04	0.34 ± 0.03	1.01 ± 0.15	15	8	Fig. S1E, F
Cdh2$^{-/-}$: αVβ3	1.95 ± 0.09	2.01 ± 0.12	0.31 ± 0.03	0.30 ± 0.04	1.05 ± 0.20	17	7	Fig. S1E, F
MZα5$^{-/-}$: αVβ3$^{\Delta 699-701}$	2.08 ± 0.13	2.12 ± 0.11	0.26 ± 0.05	0.26 ± 0.04	1.12 ± 0.16	16	14	Fig. S1H, I
MZα5$^{-/-}$: αVβ3^{AAA}	2.04 ± 0.09	2.10 ± 0.13	0.28 ± 0.03	0.27 ± 0.05	1.21 ± 0.25	19	13	Fig. S1H, I
MZα5$^{-/-}$: αVβ3^{NNNT}	2.09 ± 0.14	2.11 ± 0.13	0.26 ± 0.05	0.26 ± 0.04	1.15 ± 0.12	10	7	Fig. S1H, I

MC: mesenchymal cells; SB: somite boundaries; pos: Integrin α5-Aqm-mCit; neg: co-expression of mem-Aqm and mem-mCit.

a. *co-injected with unlabeled Integrin β3

b. Parentheses in MZα5$^{-/-}$: αVβ1, MZα5$^{-/-}$: αV^{GAANR}β3, and MZα5$^{-/-}$: αV^{GAANR}β6 indicate BiFC allele measurements of the SB/MC ratio.

Table S2. A Partial List of Proteins identified via Co-Immunoprecipitation and Mass Spectrometry, related to Figure 3.

genes	Protein Name	Accession Number	median normalized iBAQ (miBAQ)				Cluster
			$\alpha5$	αV	$\alpha V\beta3$	ctl	
fn1b	Fibronectin 1b	A2CEW3_DANRE	0.36 ± 0.34	1.23 ± 0.8	0.24 ± 0.13	-	A
lamc1	Laminin subunit gamma-1	LAMC1_DANRE	0.44 ± 0.41	1.71 ± 0.48	0.56 ± 0.66	-	A
lama1	Laminin, alpha 1	A0A0R4II20_DANRE	0.24 ± 0.17	0.92 ± 0.74	0.33 ± 0.41	-	A
apoeb	Apolipoprotein Eb	APOEB_DANRE	-	0.75 ± 0.66	-	-	A
fbn2b	Fibrillin 2b	A0A0G2KQ62_DANRE	-	0.51 ± 0.52	-	-	A
comp	Cartilage oligomeric matrix protein	A0A0G2KDJ5_DANRE	-	0.77 ± 0.18	-	-	A
itgb5	Integrin beta	F1QF91_DANRE	-	28.59 ± 9.94	6.46 ± 2.58	-	A
itga6a	Integrin, alpha 6a	A8WHQ7_DANRE	-	0.19 ± 0.07	-	-	A
efemp2b	EGF-containing fibulin extracellular matrix protein 2b	Q5WQV6_DANRE	-	1.55 ± 1.15	-	-	A
thbs3b	Thrombospondin-3b	TSP3B_DANRE	-	0.17 ± 0.08	-	-	A
thbs4b	Thrombospondin-4-B	F1R1P9_DANRE	-	0.21 ± 0.24	-	-	A
col14a1a	Collagen, type XIV, alpha 1a	F1R9Y1_DANRE	-	0.08 ± 0.02	0.04 ± 0	-	B
itgav	Integrin, alpha V	B8JLK8_DANRE	-	230.61 ± 15.4	904.97 ± 146.8	-	C
itgb3b	Integrin beta	B3DIP9_DANRE	-	-	490.47 ± 92.36	-	C
itga5	Integrin alpha5	Q5I2A9_DANRE	879.01 ± 464.01	-	-	-	D
itgb1b	Integrin beta	A0A0R4IR57_DANRE	51.1 ± 22.93	9.97 ± 0.37	1.98 ± 1.48	0.4 ± 0.1	D
itgb1a	Integrin beta	F1QG85_DANRE	26.34 ± 14.65	3.91 ± 0.7	-	-	D
fn1a	Fibronectin 1a	B0S602_DANRE	1.97 ± 1.37	1.55 ± 1.19	0.47 ± 0.57	-	D
gpc4	Glypican 4	F1QKN6_DANRE	0.19 ± 0.06	-	-	-	D
lamb1a	Laminin, beta 1a	A0A0R4IDN1_DANRE	1.35 ± 0.49	3.08 ± 0.72	1.05 ± 1.18	0.03 ± 0.02	E
col18a1a	Collagen type XVIII alpha 1 chain a	B0S8G4_DANRE	0.1 ± 0.03	-	0.08 ± 0.01	-	F

A Table of the proteins listed in Figure 3A. See full list in Table S3: The Complete List of Proteins Identified by Co-Immunoprecipitation and Mass Spectrometry.
